# The Way to Increase the Motor and Sport Competence Among Children: The Contextualized Sport Alphabetization Model

**DOI:** 10.3389/fphys.2019.00569

**Published:** 2019-05-16

**Authors:** Sixto González-Víllora, Manuel Jacob Sierra-Díaz, Juan Carlos Pastor-Vicedo, Onofre Ricardo Contreras-Jordán

**Affiliations:** EDAF Group, Didactics of Musical, Plastic and Physical Education Department, Faculty of Education, University of Castilla-La Mancha, Cuenca, Spain

**Keywords:** Small-Sided and Conditioned Games (SSCGs), physiological performance and education, child physical development, futsal, sport literacy, Models-Based Practice (MsBP)

## Abstract

There is a concern to implement games that will be able to increase the students’ *motor and sport competence* during the sport contents in Physical Education. Some games encompassed in Models-Based Practice (MsBP) are more beneficial for physical and physiological development than others. The main purpose of this study is to compare the degree of physical and physiological performance in several futsal games that have been implemented through two MsBP: the *Teaching Games for Understanding* (TGfU) and the *Contextualized Sport Alphabetization Model* (CSAM). The second objective is to analyze the relationship between physical and physiological variables. A quasi-experimental and cross-sectional study with pre- and post-test evaluations had been carried out. The sample was composed of 112 Primary Education students from First to Sixth grade (9.35 ± 1.76 years). Polar Team Pro^®^ technology was implemented to compare and analyze the physical and physiological variables. Data was analyze comparing both models with a two-step cluster model. Afterward, Student’s *t*-test was executed to compare the progression of both models. Besides, two-level multilevel model (MANOVA-ANOVA, followed by MANCOVA- ANCOVA) were also executed by means of applying a 4 versus 4 Small-Sided and Conditioned Game (SSCG). Finally, Pearson correlation between physical and physiological variables was calculated. Results showed that physical and physiological performance was higher in CSAM groups. In this regard, throughout the intervention of both models, results showed significant differences in physical and physiological variables at SSCGs implemented in the CSAM over the games implemented during the TGfU. Additionally, multilevel and MANCOVA post-test analyses shows significant differences in the physical and physiological performance during the post-test 4 vs. 4 SSCG at the CSAM students, in contrast to the TGfU students (*p* < 0.001). These results demonstrate that both physical (e.g., distance covered) and physiological performance (e.g., Edwards’ TRIMP) are significantly higher during CSAM in contrast to TGfU. Moreover, relationship between physical and physiological variables help teachers to adapt sessions to the features of the context.

## Introduction

There is a concern about the acquisition of fundamental motor skills that enables children to participate in physical activities satisfactorily (Barela, 2013). Indeed, the majority of curriculums around the world establishes that Physical Education (PE) is the most important subject that facilitate the development of the *motor competence* at the early childhood (from 6 to 12 years old). According to Holfelder and Schott (2014), this competence is defined as the capacity of executing a coordinated wide range of gross and fine motor skills. In fact, *motor competence* is closely related to *perceived motor competence*, which is the self-physical concept that inference in the level of physical activity (Utesch et al., 2018).

Particularly, PE has an important curricular part focused on the introductory stage of sports alphabetization learning or sports literacy (Kirk et al., 2006). Apart from the *motor competence*, the sports literacy contents also contribute to the acquisition of a specific competence, which enables students to solve a wide range of tactical/technical problems during their sports practice, called *sports competence* (Kolovelonis and Goudas, 2018). Nevertheless, pedagogical strategies are needed in both PE and extracurricular context to design suitable lesson plans or sessions to consolidate the tactical/technical elements, which included decision-making and skill-execution, respectively (Metzler, 2017). Therefore, rooted in the ideas of implementing Pedagogical Models (Haerens et al., 2011), whichever highlight the interdependence of the most important aspects of sport pedagogy (i.e., context, content, and teaching/learning process), Casey and MacPhail (2018) proposed the term Models-Based Practice (MsBP). This new concept aims to guarantee a contextualized and meaningful sport learning based on real practice.

On top of that, in spite of the fact that MsBP shared common features, such as the acquisition of *motor and sport competences*, they are divided according to their specific contents and didactic strategies that reinforce important aspects of the sport literacy. Hence, MsBP based on the teaching tactical/technical intelligence of the game, such as the Teaching Games for Understanding (TGfU; Morales-Belando et al., 2018), are included in the Games-Centred Approach (GCA; Harvey and Jarrett, 2014). According to Pill (2016), this framework is mainly composed by some modifications of Small-Sided and Conditioned Games (SSCGs) to emphasize a particular tactical and motor skill learning inside the game, as well as a to promote critical thinking by using discovery style through questions promoted by the teachers/coaches.

Specifically, TGfU aims to develop competent students who will be able to apply their tactical/technical knowledge properly according to each specify moment of the game, increasing gradually their autonomy (Harvey et al., 2018). The structure of this model are mainly divided into six parts (Metzler, 2017). First, the session starts with the implementation of a Modify Game. After the practice, pupils are asked about important tactical problems that they have just experienced during the game, as well as the best way to solve them. Thirdly, technical skill exercises are implemented in order to practice some technical skills related to the implemented game. After the exercises, students are asked about the most important aspects to focus on the technical ability (technical awareness). Afterward, the Modify Game is implemented again emphasizing the tactical and technical aspects learned and practiced throughout the session. Finally, a general reflection of the most important outcomes is carried out to conclude the session.

However, Kirk (2017) showed some limitations in the implementation of the TGfU in the educational context: (I) the important tension between prescription and adaptation, (II) the complex teacher preparation of a flexible and adaptable lesson plans attending each students’ needs and (III) the restriction of active participation during the questions moments. For this reason, Kirk (2017) proposed the design of a new model, which overcome the limitations observed meanwhile it adapt to the teacher and student necessities: the *Contextualized Sport Alphabetization Model* (CSAM). This new model aims to develop intelligent players who demonstrate cognitive (tactical), physical (technical) and social skills that enable them to gradually evolve toward more complex game formats.

On account of the limitations that have been found in the TGfU, Kirk (2017) highlighted that this new model should integrate some critical elements (inherent to the model), whichever are: (I) the pedagogy strategy based on the student-centered approach as well as the adaptation to the context; (II) the implementation of SSCGs adapted to the characteristics of the students, teacher and context; and (III) the holistic assessment which included contextual, small-group/team as well as individual criteria to ensure an integrate sports learning. Since each student has a different sport background, this new model is designed to have a flexible structure. On the one hand, the less skilled students learn, practice and consolidate the most basic tactical/technical intelligence. On the other hand, the students with an important sport background learn, practice and consolidate advanced contents using SSCGs.

Otherwise, it is observed a lack of investigation in the physiological responses during the implementations of MsBP at PE classes (Edwards et al., 2018). Above all, physiological scientific literature tends to focus on some physiological responses in high level performance SSCGs context such as the cardiorespiratory system and the exercise economy (Halouani et al., 2014), or the heart rate (HR) responses (Clemente et al., 2017). Furthermore, a wide range of research is focus in youth soccer athletes in an extracurricular context (Gäbler et al., 2018). For that reason, the lack of investigation about physical and physiological performance could be a limitation when MsBP are evaluated to be implemented in the educational context as a recommended way to increase the *motor and sport competence* (Goodyear et al., 2016).

Essentially, McLaren et al. (2017) observed that the training load (TL) has the potential to guarantee significant and adapted training sessions in team sports. Moreover, Paulson et al. (2015) also observed that the TLs help coaches or physical experts to avoid injury risks. The TLs are divided into two dimensions: (I) the external TL dimension, related to the physical demand stimulated by the athlete (i.e., distance, speed, or power); and (II) the internal TL dimension, related to the physiological and biochemical responses (i.e., metabolic, neurological, and cardiovascular systems responses; Vanrenterghem et al., 2017). Impellizzeri et al. (2005) spotlit that the external TL is the main factor that determines the internal TL. Besides, the TLs are a great indicator for understanding the dose-response relationship between the training and the athletes’ adaptation (Akubat et al., 2014). With regards to this, the internal TL is an important measurement to prevent both under- and over-training, as well as to achieve the desired athletes’ performances during match and training sessions (Akubat et al., 2018).

In fact, there are a myriad of invasive and non-invasive methods to evaluate the external TLs, such as the distance covered, the body load or the number of accelerations (Buchheit and Simpson, 2017); as well as the internal TLs, such as the low-frequency fatigue, the HR frequency, the lactate levels or the session-Rate of Perceived Exertion (Heishman et al., 2018). Therefore, it exists many integrated measures to quantify the TLs (Sanders et al., 2017). One of the most popular methods, proposed by Banister et al. (1975), is the training impulse (TRIMP). This method integrates the training load duration, the mean HR of the whole training session and the intensity of the exercise. Lately, Edwards (1993) proposed a modification of the formula using five arbitrary HR zones. All in all, evidence support the use of the quantification of the training loads using HR (Hoff et al., 2002; Castagna et al., 2011).

According to Jaspers et al. (2018), the implementation of monitoring technology enables coaches, physical trainers, teachers and researchers to obtain objective and reliable results using non-invasive methods. Indeed, it is also observed that the real-time data, as well as the clarity of obtaining some of the aforementioned measurements in a “friendly” way, enables coaches and teachers to design more enriching sessions, optimizing the athletes’ performance (Malone et al., 2017). Although the way of monitoring and collecting the TLs depend on the manufacturer, it is essential to use validated devices such as Polar^TM^ or Wimu^TM^ (Molina-Carmona et al., 2018) in order to obtain reliable results which could be used to customize the sessions and to improve the performance confidently. In addition, recent studies have analyzed physiological variables using monitoring technology in invasion games. In the soccer context, Rojas-Inda (2018) assessed the internal and external workload among young soccer athletes during SSCGs. Besides, Scott and Lovell (2017) analyzed the soccer training using external (GPS) and internal loads (HR and ratings of perceived exertion) among female soccer players.

However, as it has been exposed above, some limitations have been observed in the use of monitoring technologies in the educational context (i.e., expensive and complex devices). Even though the mentioned disadvantages, a new research line is focusing on quantifying the physical activity using accelerometers devices during the implementation of MsBP in Primary Education (Rocamora et al., 2019). However, to our knowledge, there is no studies focus on analyzing TLs during the models implementation in educational context.

For all the above, two objectives have been proposed in the present study. The main objective of this investigation is to determine and compare the degree of physical performance, including external TLs (i.e., distance covered, speed and number of sprints), as well as the physiological factors, including internal TLs (i.e., HR variables, integrated measurement Edwards’ TRIMP and calories), presented in several futsal sessions during the implementation of two MsBP: the TGfU and the CSAM. The second objective is to evaluate the relationship between external and internal TLs. The first hypothesis of this research indicated that when the game is properly adapted to the necessities of the students, an increase of physical and physiological aspects would be produced in contrast to general games for all the class. The second hypothesis stated that the performance global indicator Edwards’ TRIMP are positively correlated to the external TL distance covered.

## Materials and Methods

### Study Design and Variables Under Study

In order to achieve the objective of the current study, a quasi-experimental and cross-sectional study has been carried out in the educational context. In addition, pre-test and post-test evaluations were also implemented in order to determine the evolution of the physiological variables during the application of both models (Hernández-Sampieri, 2016).

**FIGURE 1:**
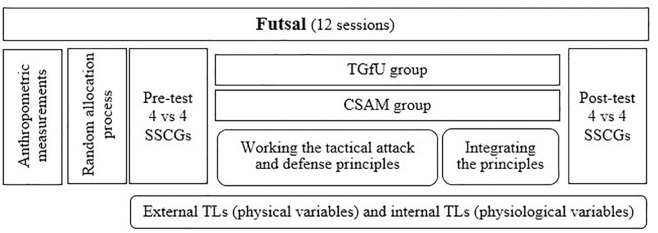
Scheme of the study design and data collect protocol.

Figure 1 summarizes the study design. Even though the content applied in each grade was the same (i.e., tactical attack and defense principles), the lesson plans were designed according to the main features and pedagogical strategies of the TGfU and CSAM. The participant allocation in each group, based on the models, was randomized. After the anthropometric measurements, the implementation of both models was applied throughout 12 sessions of 135 min per week monitoring the physical and physiological variables. In addition, 4 vs. 4 SSCGs called “mini-futsal” was carried out at the beginning and the end of the units as pre-tests and post-tests.

The independent variables included the type of models (i.e., TGfU and CSAM) and the academic grade. In this sense, Spanish education curriculum established six mandatory grades. The first grade correspond to under-seven (U-7) years old students, second grade to under-eight (U-8), third grade to under-nine (U-9), fourth grade to under-ten (U-10), fifth grade to under-eleven (U-11), and sixth grade corresponds to under-twelve (U-12) years old students. On the other hand, the dependent variables included the physical performance variables (external TLs; e.g., total distance covered) and the physiological response variables (internal TLs; e.g., Edwards’ TRIMP). In addition, anthropometric measurements, including height, weight, and waist circumference was carried out in order to contextualize the sample.

### Sample, Random Allocation, and Ethical Requirements

The sample under study was composed by 112 Primary Education (6–12 years old) male and female students from First to Sixth grade (mean age: 9.35 ± 1.76) from a State School in Cuenca, Spain. The distribution of the participants by courses was: 17.9% from First grade (*n* = 20; mean age: 6.95 ± 0.38), 17.0% from Second grade (*n* = 19; mean age 7.79 ± 0.29), 17.0% from Third grade (*n* = 19; mean age: 9.05 ± 0.41), 18.8% from Fourth grade (*n* = 21; mean age 9.91 ± 0.42), 13.4% from Fifth grade (*n* = 15; mean age: 11.11 ± 0.57) and finally, 16.1% was from Sixth grade (*n* = 18; mean age: 11.84 ± 0.27). As is it observed in Table 1, each grade was divided randomly into two groups (i.e., TGfU and CSAM group), following the CONSORT 2010 statement (Schulz et al., 2010). In each grade, every student was assigned a random identification number. The numbers were tabulated into a statistical spreadsheet. One external research from the Faculty of Education execute the random assignment command in order to obtain randomized TGfU and CSAM groups.

**Table 1:** Distribution of the sample into the two models by academic grade.

	TGfU group			CSAM group		
		
Distribution	Percentage (%)	Frequency	Mean age	Percentage (%)	Frequency	Mean age
First grade	18.18	*n* = 10	6.96 ± 0.46	17.54	*n* = 10	6.94 ± 0.30
Second grade	16.36	*n* = 9	7.77 ± 0.33	17.54	*n* = 10	7.81 ± 0.26
Third grade	16.36	*n* = 9	9.07 ± 0.46	17.54	*n* = 10	9.04 ± 0.39
Fourth grade	18.18	*n* = 10	9.73 ± 0.50	19.30	*n* = 11	10.08 ± 0.24
Fifth grade	12.73	*n* = 7	11.15 ± 0.42	14.04	*n* = 8	11.08 ± 0.70
Sixth grade	18.18	*n* = 10	11.77 ± 0.23	14.04	*n* = 8	11.90 ± 0.30
Total	100	*n* = 55	9.35 ± 1.75	100	*n* = 57	9.35 ± 1.78


Throughout the investigation, the ethical standards of the Declaration of Helsinki (World Medical Association, 2013) were exhaustively followed. In addition, all the experimental procedures and the ethical considerations were approved by the Faculty of Education of Cuenca from the University of Castilla–La Mancha (UCLM). First of all, written consent had been elaborated according to the requirements of Faden et al. (1986). Secondly, after a meeting with all the members of the school (management team, teachers, and parent delegates), the head of studies, the principal and the PE teacher give their approval to carry out the investigation in the school. Finally, the participants’ parents or participants’ legal guardians signed a written consent. Besides, all the participants voluntarily participated during the study. The privacy and the confidentiality of the participants’ personal information were exhaustively protected.

### Instruments and Materials

In order to facilitate the interpretation of the results, the implemented instruments and materials in this study have been divided into three categories.

The first category is concerning the anthropometric measurements. An exhaustive protocol was designated in order to minimize the external influence (e.g., skin temperature or hydration status) in the body composition measurement (Pinto, 2012). Primarily, height and weight values were measured twice with a 5-min interval between measurements. Height was measured using to the nearest millimeter using the calibrated stadiometer SECA^TM^ Model 213 (SECA, Corp., Hamburg, Germany). Weight, fat mass and lean mass was measured to the nearest 100 g using the bioelectrical impedance analysis system TANITA DC – 430 MA^TM^ (TANITA, Corp., Tokyo, Japan; Elia, 2013). Based on the protocol, students must not have perform any kind of intense physical activity in the last 24 h, they also must not consume soft drinks (e.g., Coca-Cola^TM^ or Fanta^TM^) or sugared drinks (e.g., orange juice) 30 min before, and they also have to urinate at least 30 min before the bioimpedance measurement. Both objective measurements were used to calculate the Quetelet Index or the Body Mass Index (BMI) as weight (in kilograms) divided by the square of the height (in meters). Then, the waist circumference was measured with a flexible tape in centimeters at the natural waist (which is the midpoint between the last rib and the iliac crest).

The second category is related to the monitoring devices, Polar Team Pro^TM^ hardware and software (Polar Electro, Corp., Finland), used to collect, in an objective way, all the physical and physiological values during the implementation of the MsBP. The main important part of this technology is (I) the Polar Team Pro^TM^ Sensor, which incorporates an integrated GPS (10 Hz), a sensor for the HR frequency, and three microelectrical mechanical components system (i.e., accelerometer, gyroscope, and digital compass; 200 Hz). This sensor has to be used with the Polar Team Pro^TM^ soft strap. The total weight of which was 60 g. In addition, sensors are chargeable using a lithium polymer rechargeable battery station called (II) Polar Team Pro Dock^TM^. Lastly, (III) the Polar Team Pro App^TM^, compatible with iPad^TM^, was used enabling the collection of the real-time data of each student. Before starting the investigation, the profiles of each student participated in the research had been created in the Polar App^TM^, including information about the height, weight, VO_2_ max (Patterson et al., 2018) and birthdate. During the implementation of the MsBP, each student wore a sensor around the chest. In fact, it was possible to extract the following physical and physiological variables with this system: the total distance covered; the distance per minutes; the maximum speed threshold (2.8 m/s^2^); the average speed; the number of sprints; the maximum, minimum, and average HR; the time in each of the five HR zones (i.e., very light: 50–60%, light: 60–70%, moderate: 70–80%, hard: 80–90%, maximum: 90–100%); and the calories burned. Goodie et al. (2000) validated the use of Polar^TM^ technology in research contexts. In addition, Polar Team Pro^TM^ was used in this investigation due to it is a device that can be easily adaptable to childhood (the size XS and S soft straps were used) and manage to collect all the data in a precise way in contrast to other devices made for adult population (Belton and MacDonncha, 2010).

The third category is regarding the method used to extract, the estimation of the VO_2_ max, as well as the Edwards’ TRIMP values. Due to the complexity of obtaining the exact VO_2_ max in the educational context, the recommendation of implementing the multistage 20-m shuttle run test (Léger et al., 1988) was taken into account. In this test, students are required to run back and forth on a 20 m track. They have to touch the 20 m line before the sound signal of a prerecorded track was emitted. However, the frequency of the sound signals increases in such a way that running speed is increased by 0.5 km per hour each minute. The starting speed is set at 8.5 km per hour. Each student’s test finishes when student is not able to follow the set pace. In the educational context, an extra session was previously implemented in order to explain this test (Ruiz et al., 2011). In addition, at the beginning of the test, a researcher also performed the test as a model together with the children to guide them. According to Lang et al. (2018) and Patterson et al. (2018) the VO_2_ max could be estimated from the number of stages (periods) of the test and the age of the students with the formula:

$${\mathrm{VO}}_{2}\;\max=31.025\;+\;3.238\;\cdot \;\mathrm{stage}\;-\;3.248\;\cdot \;\mathrm{stage}\;+\;0.1536\;\cdot \;\mathrm{stage}\;\cdot \;\mathrm{age}$$

On the other hand, in order to obtain an internal TLs global indicator of the HR, Edwards’ TRIMP was calculated using the following formula:

Edwards’ TRIMP = (minutes in maximum HR zone · 5) + (minutes in hard HR zone · 4) + (minutes in moderate HR zone · 3) + (minutes in light HR zone · 2) + (minutes in very light HR zone · 1).

### Procedure Protocol

#### Antropometric Measurements

The previous week of starting the implementation of both models, the height, weight, and waist circumference were measured following the bioimpedance body composition protocol (see section “Instruments and Materials”; Pinto, 2012) in the school gymnasium, in order to contextualize the sample, and to configure the Polar Team Pro^TM^ sensors. In addition, the 20-m shuttle run test (see section “Instruments and Materials”) was implemented in each grade.

#### Random Allocation and Pre-test SSCGs

In the first session of the model, the students in each grade were divided into the TGfU group and the CSAM group (see Table 1) following the randomization procedure (see section “Sample, Random Allocation, and Ethical Requirements”). In this first session, a pre-test 4 vs. 4 futsal SSCG was implemented in order to assess the initial level of the physical and physiological aspects. This SSCG is called “mini-futsal,” and it is played on a pitch with dimensions of 30 m × 40 m (Tavares, 2015). The objective of the game is to score a point in the goal of the other team. However, in order to reinforce the active participation of every student, each player of the team should hold or controlled the ball at least once before shooting the ball. Each SSCGs lasts 5 min in one-isolated period. There are no goalkeepers.

The implementation of both MsBP were developed in 12 sessions of 45 min each at the same time during the classes of PE. The content implemented in both models and in every grade were futsal, an extensively sport in Spanish students. Both models were implemented at the same time in the same class in order to avoid biased data. In fact, the school sport-center was divided into two parts. In each part, one model was implemented.

#### The TGfU Group

The TGfU groups followed the structure of session proposed and adapted by Metzler (2017): (I) implementation of a Modify Game, (II) common tactical awareness of the important aspect of the previous game through guided questions; (III) common technical execution and reinforcement of some technical skills; (IV) technical awareness through guided questions; (V) implementation of the Modify Game, stimulating the technical and tactical aspects developed before; and (VI) common and final reflection on the session.

In general terms, three sessions were dedicated to learn and practice each of the three tactical attack principle [(I) keep the possession of the ball, (II) progress to the rival goal, and (III) achieve the goal] and defense principle [(I) recover the possession of the ball, (II) avoid the progression of the rival team, and (III) defense the goal]. In addition, three sessions were dedicated to integrate all of the tactical principles with the best technical skill solutions. Throughout the implementation of these sessions, the students in each Modify Games were randomly changed.

The school PE teacher, an expert in the implementation of this model (more than 60 h of theoretical and practical training), designed the lesson plans together with two authors of the present research (SGV and MJSD) the TGfU lesson plans. He also was trained to implement the lesson plans in every grade, using the aforementioned structure (Metzler, 2017), by the researchers of the current paper. During the implementation, he was supervised by an external researcher (SGV).

#### The CSAM Group

On the other hand, sessions of the CSAM were adapted to the necessities to the students. Even though the CSAM do not have a “non-negotiated” structure, the sessions included (I) a beginning common reflection, (II) implementations of several SSCGs oriented to a one of the basic tactical principles of attack and defense, (III) progression of the game and reinforcement of some tactical aspect of skill of the game, and (IV) final reflection and self-evaluation. Each student in this model had to bring tracking sheet. This sheet (different in each grade) is organized in several levels (i.e., basic, intermediate, and advance) in order to facilitate the adaptation of each student’s necessities and the establishment of the individual and collective objectives.

In this model, the group was divided into several teams or sub-groups. In contrast to the TGfU, the students in each team was maintained during the first classes. The session starting with 5-min common reflection in order to establish the objectives of the session in each team (i.e., to practice the dynamic passes in a SSCGs which reinforce the idea of not seeing the ball – from a third grade tracking sheet). Thereby, each team was proposed to implement different SSCGs to fulfill the objectives that had been proposed.

Instead of implementing common tactical awareness outside the game context, as TGfU did, the reflection of the most important tactical and technical aspects was carried out through “freezing the game.” At the end of the class, a 5-min reflection of the session was implemented, in order to check if the individuals and team objectives has been achieved, and to analyzed the best outcomes of the session.

One author of the present paper (MJSD), who is also a PE teacher, designed together with the rest of the researchers (SGV, JCPV, and ORCJ), the lesson plans for this group. All authors were experts in the implementation, assessment and investigation of MsBP with more than 30 years of practice. In addition, an external researcher (SGV) also supervised the implementation of this model.

#### Data Monitoring and Post-test SSCGs

During each session, the Polar Team Pro^TM^ technology collected the real-time physical and physiological data of each student. In addition, one external researcher (SGV) was present in every session in order to supervise the implementation of each model and the recording of the data.

At the end of the implementation of both models, the 4 vs. 4 SSCG called “mini-futsal” was carried out again to compare differences between physical and physiological variables during a contextualize game, as well as to assess the improvements of each variable in each model. The students of each team were the same as those of the pre-test 4 vs. 4 SSCGs.

### Data Analysis

First of all, in order to evaluate the normal distribution of the data, a Kolmogorov–Smirnov (K–S) test was carried out due to the size of the sample (*n* = 112). The ordinary distribution is established when the mentioned test have a *p*-value superior to 0.050.

Secondly, in order to identify and assess the group of cases that share similar characteristics, the two-step cluster analysis was calculated. In this sense, this analysis could be useful to determine the quality of the allocation of the both intervention groups, identifying groups of participants that would not have been considered at the beginning of the intervention. The final clusters were compared using X^2^ test and cross-tabulations to determine if significant dependences existed (*p*-value inferior to 0.050). In addition, *k* Kappa was also calculated to evaluate the level of agreement between the clusters and the intervention groups (Landis and Kock, 1977). Once the clusters and intervention groups were evaluated, a descriptive analysis was implemented in order to compare the anthropometric variables between grades and groups (i.e., TGfU and CSAM group). These results has been reported as a mean ± standard deviation (SD) following by the *p*-value, which is significant if it is inferior to 0.050.

Thirdly, Student’s *t-*test was executed to evaluate the external and internal TLs variables monitoring during the 12 sessions (i.e., distance covered, m⋅min^-1^, maximum speed, average speed, number of sprints, maximum HR, minimum HR, average HR, Edwards’ TRIMP and calories burned) between the TGfU and the CSAM. In addition, the effect size was also calculated between both groups. The significant results were establish when *p*-value was inferior of 0.050. In addition, the effect size was also calculated between both groups.

Then, a multi-level model analysis was carried out to determine the existence of relationship between the dependent variables and the groups (i.e., TGfU and CSAM groups), including the academic grade. In this sense, *ρ* Intraclass Correlation Coefficient (ICC) was calculated.

Subsequently, MANCOVA and ANCOVA were carried out to compare the evolution through the time of the aforementioned variables derived from the same 4 vs. 4 SSCGs implemented after the investigation in every group. The significant results were established when *p*-value was inferior to 0.050. In addition, the effect size was also calculated between both groups.

Finally, on the one hand, Pearson correlation was implemented in order to evaluate the relationship between the internal TLs (i.e., total distance covered, meters per minutes -m⋅min^-1^-, maximum speed, average speed, and number of sprints) and the external TLs collected (i.e., maximum HR, minimum HR, average HR, Edwards’ TRIMP, calories burned and VO_2_ max). Alongside the Pearson’s *r* coefficient and *p*-value, it is provided the confidence intervals (CIs; Thompson, 2007) for each correlations coefficient. In addition, according to Hopkins et al. (2009), the correlation coefficient were classified in trivial (*r* from 0 to 0.09), small (*r* between 0.10 and 0.29), moderate (*r* between 0.30 and 0.49), large (*r* between 0.50 and 0.69), very large (*r* between 0.70 and 0.89), nearly perfect (*r* between 0.90 and 0.99), and perfect (when *r* is 1). On the other hand, partial correlations were executed to control the confounding influence of the third intervening variables (e.g., gender, course, and intervention groups). In both procedures, significance was set at *p*-value inferior to 0.050.

All the above mentioned statistical procedures were calculated using the Statistical Package for the Social Sciences (SPSS^TM^), version 24. The Cohen’s *d* effect size value is considered small from 0 to 0.20, medium form 0.21 to 0.50, large from 0.51 to 0.80, and very large from more than 1.30 (Sullivan and Feinn, 2012).

## Results

Since the sample was superior to 30 subjects, the normal distribution was assumed due to the Central Limit Theorem (CLT) (Akritas and Papadatos, 2004).

### Two-Step Cluster Procedure and Anthropometric Descriptive Analysis

The physical and physiological dependent variables were explored using the two-step cluster procedure. Results identify two clusters with a good cluster quality of 0.50 (Mooi and Sarstedt, 2011). Table 2 shows the analysis of the dependent variables according to the two clusters. First of all, the first cluster was comprised of 51.40% of the sample with significant physical and physiological responses due to the methodology implemented. The largest relationship group in this cluster was First grade at 19.60%. The second cluster was comprised of 48.60% of the sample. This second cluster captured students with fewer means of the physical and physiological variables. A total of 20.8% of this group came from Fourth grade. The X^2^ analysis showed statistical relationship between both clusters and the intervention groups (*X*^2^ = 97.360; *p* < 0.001), categorized as large effect (*V* = 0.945). In addition, *k* Kappa showed almost perfect agreement between both clusters and the intervention groups (*k* = 0.945; *p* < 0.001).

**Table 2:** Analysis of the significant differences between both clusters.

Clusters	1	2
% of cases	51.4%	48.6%
Distance covered^∗^	1421.68	771.85
Average HR^∗^	179.62	146.91
Number of sprints^∗^	13.81	5.96
Edwards’ TRIMP^∗^	21	11.45
Maximum HR^∗^	204.76	187.99
Minimum HR^∗^	112.25	95.99
Calories burned^∗^	142.00	86.30
m⋅min^-1∗^	52.63	30.80
Average speed^∗^	3.96	2.27


*^∗^Significant results (*p*-values inferior than 0.050).*

Secondly, Table 3 shows the average of the anthropometric values in each grade, divided by the intervention groups (i.e., TGfU and CSAM). In addition, Student’s *t*-test was carried out in order to determine significant differences between both groups. The total average did not show significant statistical differences at height (*p* = 0.653), weight (*p* = 0.588), BMI (*p* = 0.272), and waist circumference (*p* = 0.389) among groups.

**Table 3:** Anthropometrics variables divided by grade and intervention groups.

Grade (years)	Group	Anthropometric variables			
		
		Height (cm)	Weight (kg)	BMI (kg/m^2^)	Waist circumference (cm)
First (U-7)	TGfU	115.90 ± 5.27	22.09 ± 4.24	16.31 ± 1.92	54.20 ± 6.35
	CSAM	120.70 ± 3.86	23.81 ± 2.35	16.35 ± 1.56	57.40 ± 4.37
Second (U-8)	TGfU	126.80 ± 7.62	29.12 ± 8.07	17.93 ± 3.91	55.90 ± 2.84
	CSAM	127.30 ± 9.86	26.52 ± 6.06	16.18 ± 1.45	65.22 ± 10.07
Third (U-9)	TGfU	131.44 ± 7.55	29.54 ± 7.79	16.95 ± 3.40	61.44 ± 9.64
	CSAM	133.70 ± 32.44	32.44 ± 8.32	17.96 ± 3.47	66.20 ± 12.30
Fourth (U-10)	TGfU	137.20 ± 7.92	34.50 ± 9.82	18.05 ± 3.52	66.90 ± 10.47
	CSAM	136.90 ± 6.45	33.69 ± 6.47	17.85 ± 2.41	64.82 ± 7.34
Fifth (U-12)	TGfU	140.20 ± 33.08	33.08 ± 6.69	16.71 ± 2.07	60.14 ± 4.22
	CSAM	144.25 ± 10.44	41.92 ± 13.82	19.68 ± 3.79	71.50 ± 10.70
Sixth (U-12)	TGfU	149.80 ± 6.61	51.65 ± 14.80	22.79 ± 5.43	79.00 ± 16.80
	CSAM	148.25 ± 8.46	38.46 ± 9.27	17.30 ± 2.61	63.38 ± 7.42
Total average	TGfU	133.38 ± 5.27	33.49 ± 13.05	18.23 ± 4.14	64.74 ± 12.90
	CSAM	134.43 ± 11.69	32.30 ± 9.89	17.49 ± 2.76	65.93 ± 9.32


*U-x, under-age; cm, centimeter; kg, kilogram; m, meter.*

### During Intervention Analysis: Comparison of Both Models

In order to determine significant differences in the external and internal TLs during the 12 sessions between both models, Student’s *t*-test was carried out. Table 4 shows significant statistical differences between TGfU and CSAM groups. Indeed, throughout the lesson plans, better results were observed when CSAM are implemented, in contrast to TGfU, at total distance covered (*p* < 0.001), m⋅min^-1^ (*p* < 0.001), maximum speed (*p* < 0.001), average speed (*p* < 0.001), and number of sprints (*p* < 0.001). In addition, this trend was also observed at internal TLs values, which included maximum HR (*p* < 0.001), minimum HR (*p* < 0.001), average HR (*p* < 0.001), Edwards’ TRIMP (*p* < 0.001), and calories burned (*p* < 0.001). In this sense, Cohen’s *d* effect size was significantly large in all the TLs variables (Supplementary Material).

**Table 4:** Analysis and comparison of the external and internal TLs throughout the sessions.

	TGfU group		CSAM group		*p*	*d*
				
	*Mean*	*SD*	*Mean*	*SD*		
Distance covered	783.22	154.57	1422.33	212.16	<0.001	3.43
m⋅min^-1^	32.22	13.20	52.28	16.58	<0.001	1.33
Maximum speed	19.35	2.85	23.48	3.63	<0.001	1.26
Average speed	2.35	0.75	3.42	1.05	<0.001	1.20
Number of sprints	5.96	1.10	13.88	3.17	<0.001	3.33
Maximum HR	188.40	9.77	204.34	7.44	<0.001	1.83
Minimum HR	97.15	10.69	111.75	7.47	<0.001	1.58
Average HR	147.48	9.09	179.55	9.16	<0.001	3.51
Edwards’ TRIMP	11.42	3.37	21.30	2.11	<0.001	3.51
Calories burned	85.67	22.97	142.69	40.33	<0.001	1.73


*SD, standard deviation; *p, p*-value; d, Cohen’s *d* value.*

### Multilevel Model Analysis and Intraclass Correlation Coefficient

The two-level model was used to predict the physical and physiological outcomes at the 4 vs. 4 SSCGs pre-test and post-test variables using the cluster proposed in Section “Two-Step Cluster Procedure and Anthropometric Descriptive Analysis,” the groups intervention and the academic grades. Table 5 shows that the regression coefficient for pre-test variables indicated a positive and significant relationship between the corresponding pre-test variables and the post-test dependent variables. The proportions of variation in physical and physiological variables during the 4 vs. 4 SSCGs that lies between intervention groups and grades varied from 1 to 48.4%.

**Table 5:** Regression estimation of the pre-test and post-test variables using the two-level model.

Variables	Groups means differences		Regression coefficient	*SE*	*p*	ρ ICC
					
	TGfU	CSAM				
Distance covered	74.46	125.58	305.104	16.860	<0.001	0.001
m⋅min^-1^	1.77	9.96	17.980	1.172	<0.001	0.011
Maximum speed	1.86	7.96	15.316	3.585	<0.001	0.021
Average speed	0.24	1.01	0.884	0.118	<0.001	0.484
Number of sprints	3.77	9.04	6.165	0.426	<0.001	0.160
Maximum HR	25.29	52.18	120.949	20.719	<0.001	0.001
Minimum HR	18.11	31.98	54.21	10.577	<0.001	0.010
Average HR	21.56	37.35	82.772	18.153	<0.001	0.004
Edwards’ TRIMP	-0.09	10.31	14.072	1.088	<0.001	0.012
Calories burned	11.02	24.56	25.040	4.108	<0.001	0.026
VO_ 2_ max	5.92	19.62	56.419	5.260	<0.001	0.014


*ICC, Intraclass Correlation Coefficient.*

### Analysis of the Pre-test and Post-test 4 vs. 4 SSCG

In relation to the homogeneity of physical and physiological variables derived from the 4 vs. 4 SSCGs carried out at the beginning of the implementation, the MANOVA result did not show statistical differences in external TLs and internal TLs [Wilks’ Lambda Λ = 0.927, *F*(11,100) = 0.715, *p* > 0.050; very large effect size, η^2^ = 0.73] between models. Indeed, Table 6 shows that pre-test ANOVA results did not show statistical differences between TGfU and CSAM (*p* > 0.050).

**Table 6:** Descriptive and inferential pre-test analysis of the external and internal TLs variables.

	Pre-test				ANOVA pre-test		
		
	TGfU group		CSAM group		*F*(1,110)	*p*	*d*
					
	*Mean*	*SD*	*Mean*	*SD*			
Distance covered	197.96	54.22	197.89	58.90	0.0	0.99	-0.001
m⋅min^-1^	17.96	3.44	15.25	3.36	1.2	0.26	-0.797
Maximum speed	16.35	1.19	16.19	1.02	0.5	0.44	-0.144
Average speed	1.99	3.07	1.54	0.58	1.1	0.28	-0.203
Number of sprints	0.85	0.80	0.75	0.78	0.4	0.50	-0.126
Maximum HR	100.51	9.97	100.40	9.80	0.0	0.95	-0.011
Minimum HR	70.98	11.99	70.49	12.11	0.0	0.83	-0.040
Average HR	84.29	9.69	83.93	9.51	0.0	0.84	-0.037
Edwards’ TRIMP	11.67	3.73	11.19	3.55	0.4	0.48	-0.131
Calories burned	33.69	10.80	32.53	9.95	0.3	0.55	-0.111


*m, meter; min, minute; SD, standard deviation; *p, p*-value; *d*, Cohen’s *d* value.*

Subsequently, MANCOVA results showed statistical differences in external and internal TLs between TGfU and CSAM among each grade during the 4 vs. 4 SSCGs carried out after the implementation of them [Wilks’ Lambda Λ = 0.900, *F*(10,90) = 90.715, *p* < 0.001; nearly perfect effect size, η^2^ = 0.91]. In fact, Table 7 shows that post-test ANCOVA results were significantly different at CSAM in contrast to TGfU, observing a significant *p*-value in each external and internal TLs (*p* < 0.001) (Supplementary Material).

**Table 7:** Descriptive and inferential post-test analysis of the external and internal TLs variables.

	Post-test				ANCOVA post-test		
		
	TGfU group		CSAM group		*F*(1,110)	*p*	*d*
					
	*Mean*	*SD*	*Mean*	*SD*			
Distance covered	272.42	53.82	323.47	53.45	202.8	<0.001	0.951
m⋅min^-1^	19.73	3.47	25.21	3.20	102.2	<0.001	0.419
Maximum speed	18.21	2.21	24.15	2.37	281.6	<0.001	2.592
Average speed	2.23	3.01	2.55	0.36	367.2	<0.001	0.149
Number of sprints	4.62	2.92	9.79	3.81	110.0	<0.001	1.523
Maximum HR	125.80	16.18	152.58	12.31	142.6	<0.001	1.862
Minimum HR	89.09	12.76	102.47	11.10	202.8	<0.001	1.118
Average HR	105.85	19.78	121.28	13.69	169.5	<0.001	0.907
Edwards’ TRIMP	11.58	3.53	21.50	2.21	366.3	<0.001	3.368
Calories burned	44.71	14.21	57.09	13.81	146.0	<0.001	0.883


*m, meter; min, minute; SD, standard deviation; *p, p*-value; *d*, Cohen’s *d* value.*

### Relationship Between External and Internal TLs

In order to analyze the TLs as a whole, a significant relationship should be establish between external and internal TLs in educational context. The correlation between the external TLs and the internal TLs derived from the record in every session shows significantly statistical differences from small to moderated among both MsBP. Specifically, the TGfU model shows that total distance covered are significantly related to maximum HR (*r* = 0.26, *p* = 0.050, CI: 0.00 to 0.49; small), to average HR (*r* = 0.28, *p* = 0.036, CI: 0.02 to 0.51; small), and to the calories burned (*r* = 0.27, *p* = 0.042, CI: 0.01 to 0.50; small). Moreover, it is also observed a significant relationship between m⋅min^-1^ and maximum HR (*r* = 0.31, *p* = 0.018, CI: 0.58 to 0.53; moderate), m⋅min^-1^ and average HR (*r* = 0.34, *p* = 0.009, CI: 0.82 to 0.53; moderate), as well as m⋅min^-1^ and minimum HR (*r* = 0.26, *p* = 0.048, CI: 0.00 to 0.49; small). Maximum speed is also significant related to maximum HR (*r* = 0.27, *p* = 0.046, CI: 0.00 to 0.50; small). Finally it is also observed a moderated relationship between average speed with maximum HR (*r* = 0.39, *p* = 0.003, CI: 0.14 to 0.59; moderated), and average HR (*r* = 0.44, *p* = 0.001, CI: 0.23 to 0.60; moderate).

In addition, the CSAM shows that total distance covered are significantly related to maximum HR (*r* = 0.42, *p* = 0.001, CI: 0.18 to 0.61; moderate), to average HR (*r* = 0.46, *p* < 0.001, CI: 0.22 to 0.64; moderate) and to the calories burned (*r* = 0.63, *p* < 0.001, CI: 0.44 to 0.76; large). It is also observed a moderate relationship between total distance covered and Edwards’ TRIMP (*r* = 0.39, *p* = 0.003, CI: 0.14 to 0.59; moderate). This model also shows a significant relationship with number of sprints and maximum HR (*r* = 0.31, *p* = 0.017, CI: 0.05 to 0.52; moderate). Besides, the number of sprints are largely related to the average HR (*r* = 0.65, *p* < 0.001, CI: 0.46 to 0.77; large), and moderated related to the VO_2_ max (*r* = 0.39, *p* = 0.003, CI: 0.14 to 0.59; moderate).

In relation to the partial correlation, taking account the confounding influence of the gender, course and intervention groups, the magnitude of the aforementioned correlations was reduced. In this sense, on the one hand, it is observed a small correlation effect between the m⋅m^-1^ and the calories burned (*r* = 0.27, *p* = 0.005), as well as between the number of sprints and the minimum HR (*r* = 0.20, *p* = 0.032). On the other hand, it is also detected a moderate correlation effect among the total distance covered with the maximum HR (*r* = 0.31, *p* < 0.001), the average HR (*r* = 0.38, *p* < 0.001) and the calories burned (*r* = 0.46, *p* < 0.001). Moreover, it is also observed a moderate relationship among the number of sprints with the maximum HR (*r* = 0.30, *p* < 0.001), the average HR (*r* = 0.48, *p* < 0.001), and the calories burned (*r* = 0.39, *p* < 0.001).

## Discussion

There is a lack of research in the assessment of TLs in the educational context, and specifically in MsBP. The main objective of this investigation was to assess and compare the degree of physical performance and the physiological factors derived from the implementation of TGfU and CSAM, in order to determine the motor and sport competence in both models. Furthermore, the second objective of the present study is to analyze the relationship between external and internal TLs. In addition, it is confirmed the main hypothesis of this research. When the sessions and the SSCGs are properly adapted to the students’ necessities and abilities, engaging students to achieve individual and group objectives (use during CSAM), it is observed a significant increase of both physical and physiological variables in contrast to the use of general games and tactical awareness implemented for all the class (use of the TGfU). Secondly, it is confirm the hypothesis of the positive correlation between external and internal TLs.

First of all, two-step cluster analysis clearly identified two groups, similar from the intervention groups. Indeed, Kappa coefficient showed almost perfect agreement between these categories. In this sense, first clusters is closely related to CSAM group, where distance covered and HR variables are significant high in contrast to the second cluster, which is closely related to TGfU group.

Significant differences have not been observed regarding anthropometric measurements that have not been based on the intervention groups. However, in line with Silva-Arantes (2018), the anthropometric measurements should be a basic index to identify sedentary lifestyle (i.e., overweight) and to orientate more effective intervention in PE related to healthy habits. In this sense, one of the most popular interventions to control the overweight and obesity is the BMI (Khambalia et al., 2012) and the waist circumference (Fredriksen et al., 2018). In the current paper, ordinary BMI and waist circumference have been observed in both models. Similar results were observed by Brown et al. (2018), obtaining that BMI improved when the intervention is oriented to lifestyle habits. Since Nuttall (2015) observed that BMI has some limitations due to it does not differentiate between fat mass and lean mass, Elia (2013) has recommended the use of bioelectrical impedance devices to identify the kind of body mass. However, in the present study it is show normal weight, closely related to the BMI results.

Regarding the comparison of the external and internal TLs variables that are derived from the recording of all the sessions, significant differences are observed in CSAM group in contrast to the TGfU group (*p* < 0.001). Used Modify Games in TGfU are those ones that that have been adapted to the class needs. However, SSCGs formats in CSAM are games which follow a progression of the difficulty and have been adapted to the aims that have been proposed by each student in an individualized way (e.g., to work the demarcations) and the teams (e.g., to practice the possession of the ball using attacking inferiority). Similar results have been observed in comparable studies (Clemente et al., 2017; González-Víllora et al., 2017), where the small formats of the game significantly increase the maximum HR, as well as the HR frequency in each zone.

In this line, Martín-Martínez et al. (2015) highlights that the SSCGs implementation supposes an increase of the physical and physiological response, due to it, a more significant participation of the players has been observed. The current paper has consequently verified that the active participation of the students in the implementation of SSCGs during the CSAM has increased the physical performance, such as the total distance covered (*p* < 0.001), and the physiological response, such as the average HR (*p* < 0.001). According to that, the concern of Kirk (2017) has been empirically demonstrated improvements are produced in the CSAM due to the SSCGs implementation during the season have been adapted to the students needs, in contrast to the Modify Games that have been organized in several games to practice the tactical/technical attack and defense principles.

On the contrary, Nathan (2017) found that TGfU is a positive model that achieve better results to enhance intensity and HR frequency in coaching environments, in contrast to strategies that are based on technical skill-drills. Similar results were found in educational context (Li et al., 2018), skill-drills are a decontextualized sport practice belonging to traditional PE approach. Even though the TGfU was created to contextualized the sport practice at introductory stage of sport learning, the result in this study also confirm that in some cases TGfU is confused as a “prescription tool” and not a pedagogical strategy which needs context adaptations (Kirk, 2017). In this sense, models should be focus on the heterogeneity of the students and the way to satisfy their necessities at the same time as the curricular sport contents and the tactical/technical intelligence are consolidated satisfactorily.

In addition, the common tactical and technical awareness parts in TGFU (Gray and Sproule, 2011) could seem to have a negative impact on the physical and physiological responses. The current paper has demonstrated that “freezing the game” (used in CSAM) could be a better strategy to engage tactical/technical thinking (Práxedes et al., 2016), as well as to increase the active time of physical and physiological performance instead of using a tactical/technical awareness outside the game (used in TGfU). This strategy is based on short periods of reflection times, when important aspect should be considered. In this sense, tactical/technical progression of the CSAM enables a contextualize and active reflection.

In relation with the values obtaining in pre- and post-test 4 vs. 4 SSCG multilevel and variance analyses, it is observed what Cordova et al. (2012) has previously postulated, has been observed, this being: physical performance, including total distance covered, m⋅m^-1^, maximum speed threshold, average speed and number of sprints; as well as physiological responses, including HR values, Edwards’ TRIMP and the burnt calories have increased at the end of both models (*p* < 0.001). In this sense, according to Rojas-Inda (2018), it is important to collect TLs with simply methods that could be applied in educational context, such as the distance covered or the HR values to help teacher to make decision about the design of the session or the specific games.

Considering the research of Dellal et al. (2011) in an extracurricular context, and Atl et al. (2013) in Secondary Education, it is also confirmed that the implementation of SSCGs is a beneficial strategy to increase the physical response and the physiological responses during CSAM. Furthermore, the evolution of the SSCGs, according to the needs and targets of the students in CSAM, produces significant improvements in contrast to keep the same number of players at Modify Games (TGfU). In this respect, similar results are also observed in elite soccer by Olthof et al. (2018). Recently, García-Angulo et al. (2019) highlighted that using non-linear pedagogy (such as the models used in this investigation) during the implementation of SSCGs can produced significant increase of vigorous physical activity. Indeed, CSAM students perceived SSGs as the best way to practice their tactical/technical intelligence focusing on achieving common objectives as a team inside the game, and helping other students to achieve their personal objectives proposed at the beginning of each sessions.

Regarding the relationship between the TLs, positive correlations obtaining in the present in some of the external TLs variables (i.e., total distance covered, m⋅m^-1^, maximum speed, average speed and number of sprints) with some of internal TLs variables (i.e., maximum, minimum, and average HR; calories burned; and VO_2_ max) are observed. On the one hand, this relationship confirm the idea that external TLs are the factors whichever determine the internal TLs. On the other, this fact confirm the main idea of Akubat et al. (2014), who showed that the integration of both TLs during the implementation of the MsBP is more useful than registering only external TLs. In this respect, these results support the idea of Kirk (2017), which highlighted that pedagogical strategies should be implemented according to the real necessities and abilities of the students. In fact, monitoring devices help to obtain real-time data from all the students in order to adapt the SSCGs to the students’ characteristics and sport literacy objectives (Malone et al., 2017; Rocamora et al., 2019).

In addition, results showed in the meta-analysis by McLaren et al. (2017) highlighting that the relationships of external and internal TL could be different depending of the mode of training. This fact is also confirmed in the present study in the educational context. The relationship between external and internal TLs is different among TGfU students from CSAM students. Bartlett et al. (2017) confirmed that the total distance covered have the strongest association with internal TL in team-sports. Indeed, the correlation observed during the introductory stage of team-sport learning context in PE, seems to follow the same trend: the distance covered is significantly related with the HR values. For that reason, these results seem to reaffirm the idea observed in extracurricular sport context (Scanlan et al., 2014): external TLs, such as the total distance covered or the maximum speed threshold, seem to influence the internal response of the players.

Particularly, correlation between distance covered and Edwards’ TRIMP could be only observed in the implementation of CSAM (*r* = 0.39, *p* = 0.003). Even though the lack of findings in educational context, similar results was found by Casamichana et al. (2013) in soccer. In this study, it is observed a large correlation between distance covered and Edwards’ TRIMP. Finally, Haddad et al. (2017) and Fitzpatrick et al. (2018) highlight that the relationship between TLs help to examine the dose-response relationship, which is important to effective training programs. In this sense, Casamichana et al. (2013) observed that HR based methods using validated real-time devices (Malone et al., 2017) will help to objective track the internal TLs.

Nevertheless, the results should be taken cautiously due to new investigations in this area as well as models are needed. Even though the sample was composed by 112 students from every grade of Primary Education, more sample will be needed to consolidate the results observed in the present study attending to each educational context. Indeed, future research should be taken into account the comparison of dependent variables using a control group. In addition, this research investigated the physical and physiological variables, which are a group of values very important to assess new pedagogical strategies at PE or at team sport context.

However, it should be interesting to evaluate the tactical/technical knowledge as well as psychosocial factors in order to obtain a holistic evidence of the MsBP. In fact, new research could be oriented to evaluate external and internal TLs in educational context, as well as to correlate the Session-Ratings Perceived Exertions TLs with other external TLs such as the accelerometer load of the number of sustained impacts. Therefore, future research will be focus on the teacher perception when designs and prepares this innovative model, as well as the level of training and preparation so that the model could be carried out.

### Practical Applications

**FIGURE 2:**
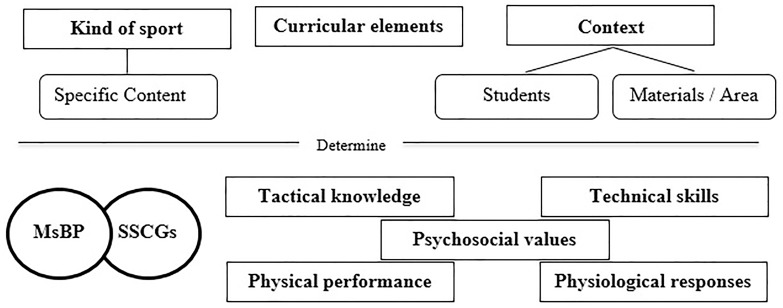
Factors that determine an effective acquisition of *sport competence* during MsBP.

Teaching/learning process at an introductory stage in sports alphabetization learning (PE and extracurricular context) is a very complex issue. For this reason, MsBP imply a great resource to orientate the pedagogical process (Casey and MacPhail, 2018). In fact, as it is observed in Figure 2, teachers should be taken into account some important aspects during the implementation of the models selected: (I) the kind of sport, which determine the type of specific content; (II) all the curricular elements (e.g., objectives, competences, or contents); and (III) the features of the context, which include (a) the grade or the years old of the group, (b) the previous knowledge of the content selected, (c) the needs and motivation of every students, (d) the specific materials, and (e) the area of play available. Indeed, these elements will determine the *motor and sport competence* acquisition during each session, which are divided into (I) tactical (decision-making) knowledge, (II) technical (skills) abilities, (III) physical performance or external TLs, (IV) physiological response or internal TLs, and (V) positive psychosocial values.

In this sense, according to Kirk (2017), the implementation of MsBP does not mean that they are “blueprints” which can be applied in every context. Particularly, the systematic review by Stolz and Pill (2014) proved that TGfU has got many versions and iterations around the world. This fact causes confusion in the educational context, where tension between prescription and adaptations is present (Kirk, 2017).

For this reason, it is important to reinforce the idea of attending to the features of each context, as well as the needs, abilities and motivations of the students and teachers. Indeed, the present study shows how CSAM could be a great resource for teachers to guide and adapt the content and the SSCGs according to the characteristics of each student, in contrast to ‘obligate’ them to adapt to the game. In this sense, teacher should know the start point of each student, as well as the time of the sport practice outside the PE classes. For that reason, tracking sheet (used in CSAM) help students to know where they are, and where they want to be in the future regarding tactical/technical intelligence. Indeed, it is important for students to perceive a positive evolution of their abilities. In this regard, close relationship between teacher and student is necessary. Furthermore, González-Víllora et al. (2018) showed that the combination of different features of the MsBP or the hybridization of them are an innovative trend to increase the *motor and sport competence* by amalgamating the basic features of each MBP.

**FIGURE 3:**
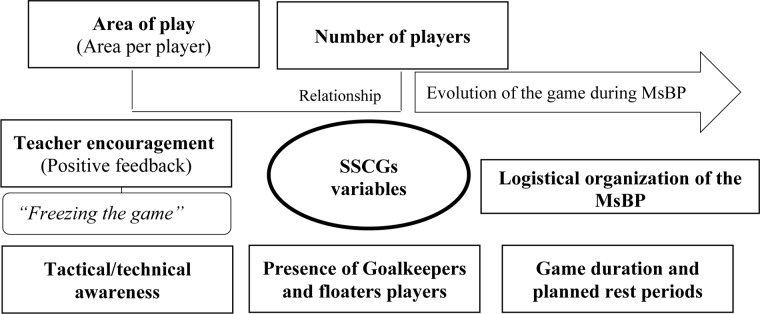
Variables that can be controlled by the teachers or coaches during Small-Sided and Conditioned Games.

One of the most important elements in the MsBP studied in the current paper is the implementation of SSCGs. According to Hill-Haas et al. (2011), these kinds of games should be the central axis of the PE sessions due to they offer many practical advantages. Clemente (2016) highlighted that SSCGs allow to replicate the physical performance and the physiological responses of the real match play, facilitating the evolution of tactical awareness and technical skills into a contextualized, and adapted MsBP and SSCGs atmosphere. Besides, it is observed that the implementation of SSCGs increases the students’ compliance and motivation (Hill-Haas et al., 2011). In this sense, Figure 3 shows that there are many variables that can be controlled by the teachers or coaches to ensure holistic development of the *motor and sport competence*, which are also complemented by some logistic variables of the MsBP (e.g., grade of the students or type of sport).

In terms of measuring the physical and physiological values, Pind and Mäestu (2017) recommend to monitor TLs using real-time devices (such as validated pulsometers and/or accelerometers), as well as other strategies to understand the internal TLs responses (such as using the Edwards’ TRIMP). Even though, these kinds of devices could not be available in the educational context, teachers should take into account indirect strategies or methods to measure the HR of their students (e.g., the PSE). These measurements enable to guide or help teachers and coaches to organize and design PE sessions adequately and confidently ensuring a harmonic student development of the *motor and sport competence*.

## Conclusion

Within an educational context, the implementation of MsBP should not only be determined by empirical pedagogical improvements and tactical/technical progresses, but also by the physical and physiological variables which also contribute to the *motor and sport competences*. For this reason, there is a need to obtain physical and physiological variables of each MsBP. Thereby, it has been highly appreciated that the implementation of CSAM enables to overcome some of the limitations of the TGfU: the nature of the contextualized constraints in this new model will help to achieve better physical and physiological results.

It is confirmed the idea that MsBP should be adapted to the circumstances of the context (including the students’ needs and motivation). Besides, the MsBP encompassed in the GCA should be organized according to an effective evolution of SSCGs. This kind of games is an effective strategy to increase physical and physiological performance when they are adapted according to the necessities and objectives of the students.

Monitoring real-time data are also ideal methods to quantify the physical performance (external TLs) and physiological responses (internal TLs) in educational context. For these reasons, there is a need to measure both external and internal TLs. Indeed, the relationship between the TLs has led teachers to ‘listen to’ the needs and abilities of each student in order to design and organize efficient MsBP sessions. Although improvements in physical and physiological variables at the end of both MsBP implementations have been observed, the CSAM groups have obtained better results in the physical and physiological variables in contrast to the TGfU groups.

In conclusion, *motor and sport competence* are closely related to the physical and physiological variables among others (i.e., pedagogical strategies, tactical/technical awareness, as well as psychosocial and prosocial values). However, the contribution of these elements to the above mentioned competence are not an intrinsic factor inside the MsBP or the SSCGs. There is a true need to ‘listen to’ the necessities and abilities of the student, as well as to design sessions according to these necessities and the context, where everybody can find his/her place inside the game, meanwhile the *motor and sport competence* effectively are being increased.

## Ethics Statement

The investigation “The way to increase the motor and sport competence among children: the Contextualized Sport Alphabetization Model” was designed based on the Ethical standards of the Declaration of Helsinki at the 64th World Medical Association (2013). The authors/researchers of this investigation have followed rigorously this Ethical Declaration due to the research involved human subjects (112 students from a Spanish State Primary School -9.35 ± 1.76 years old-). As it is shown in the Frontiers in Physiology manuscript, the study fulfilled with all the ethical standards, especially those related to the Privacy and Confidentiality (Principle 24); and Informed Consent (from Principles 25 to 32), adapted to Social Sciences and educational context (Faden et al., 1986). The informed consent before implementing the investigation was based rigorously on the manual “A history and theory of Informed Consent” (Faden et al., 1986). According to the current Spanish educational laws in force (the 9th of December Organic Law 8/2013, for the improvement of the educational quality –LOMCE-, as well as the 14th of July Decree 54/2014, which sets the curriculum for Primary Education in Castilla–La Mancha), the original informed consent signed by the head of studies, the principal, the Physical Education teacher, as well as each parent or legal guardian of the pupils should be kept in the school for future references. Furthermore, due to the audio-visual recording during the Small-Sided Games [as well as for others kind of activities during the academic year], the School had to request parental permission with an extra consent (Article 13.1 of the Organic Law of the Data Protection -LOPD-) at the beginning of the academic year. This consent has to be guarded in the school for future inspections.

## Author Contributions

SG-V and MJS-D were involved in the conception and designed of the research and performed the experiments. SG-V, MJS-D, JP-V, and OC-J analyzed the data, edited and revised the manuscript, prepared the figures, drafted the manuscript, interpreted results of the experiments, and approved final version of the manuscript.

## Conflict of Interest Statement

The authors declare that the research was conducted in the absence of any commercial or financial relationships that could be construed as a potential conflict of interest.

## References

[B1] Akritas M. G. Papadatos N. (2004). Heteroscedastic one-way ANOVA and lack-of-fit tests. *J. Am. Stat. Assoc.* 99 368–382. 10.1198/016214504000000412

[B2] Akubat I. Barrett S. Abt G. (2014). Integrating the internal and external training loads in soccer. *Int. J. Sports Physiol. Perform.* 9 457–462. 10.1123/IJSPP.2012.0347 23475154

[B3] Akubat I. Barrett S. Lapuente-Sagarra M. Abt G. (2018). The validity of external: internal training load ratios in rested and fatigued soccer players. *Sports* 6 1–9. 10.3390/sports6020044 29910348 PMC6026787

[B4] Atl H. Köklü Y. Alemdaroglu U. Ünver-Koçak F. (2013). A comparison of heart rate response and frequencies of technical actions between half-court and full-court 3-a-side games in high school female basketball players. *J. Strength Cond. Res.* 27 352–356. 10.1519/jsc.0b013e3182542674 22465987

[B5] Banister E. W. Calvert T. W. Savage M. V. Bach A. (1975). A systems model of training for athletic performance. *Aust. J. Sports Med.* 7 57–61.

[B6] Barela J. A. (2013). Fundamental motor skill proficiency is necessary for children’s motor activity inclusion. *Motriz* 19 548–551. 10.1590/S1980-65742013000300003 27293501

[B7] Bartlett J. D. O’Connor F. Pitchford N. Torres-Ronda L. Robertson S. J. (2017). Relationships between internal and external training load in team-sport athletes: evidence for an individualised approach. *Int. J. Sports Physiol. Perform.* 12 230–234. 10.1123/ijspp.2015-0791 27194668

[B8] Belton S. MacDonncha C. (2010). Reliability and validity of a new physical activity self-report measure for younger children. *Meas. Phys. Educ. Exerc. Sci.* 14 15–28. 10.1080/10913670903454994 28262647

[B9] Brown E. C. Buchan D. S. Drignei D. Wyatt F. B. Kilgore L. Cavana J. (2018). Primary school children’s health behaviors, attitudes, and body mass index after a 10-week lifestyle intervention with follow-up. *Front. Pediatr.* 6:137. 10.3389/fped.2018.00137 29868527 PMC5954212

[B10] Buchheit M. Simpson B. M. (2017). Player-tracking technology: half-full or half-empty glass? *Int. J. Sports Physiol. Perform.* 12 235–241. 10.1123/ijspp.2016-0499 27967285

[B11] Casamichana D. Castellano J. Calleja-González J. Román J. Castagna C. (2013). Relationship between indicators of training load in soccer players. *J. Strength Cond. Res.* 27 369–374. 10.1519/jsc.0b013e3182548af1 22465992

[B12] Casey A. MacPhail A. (2018). Adopting a models-based approach to teaching physical education. *Phys. Educ. Sport Pedag.* 23 294–310. 10.1080/17408989.2018.1429588

[B13] Castagna C. Impellizzeri F. M. Chaouachi A. Bordon C. Manzi V. (2011). Effect of training intensity distribution on aerobic fitness variables in elite soccer players: a case study. *J. Strength Cond. Res.* 25 66–71. 10.1519/jsc.0b013e3181fef3d3 21150673

[B14] Clemente F. M. (2016). *Small-Sided and Conditioned Games in Soccer Training. The Science and Practical Applications.* New York, NY: Springer.

[B15] Clemente F. M. González-Víllora S. Delextrat A. Lourenço-Martins F. M. Pastor-Vicedo J. C. (2017). Effects of the sports level, format of the game and task condition on heart rate responses, technical and tactical performance of youth basketball players. *J. Hum. Kinet.* 58 141–155. 10.1015/hukin-2017-0080 28828085 PMC5548162

[B16] Cordova A. Villa C. Sureda A. Rodríguez-Marroyo J. A. Sánchez-Collado M. P. (2012). Physical activity and cardiovascular risk factors in Spanish children aged 11-13 years. *Rev. Esp. Cardiol.* 65 620–626. 10.1016/j.recesp.2012.01.026 22633280

[B17] Dellal A. Hill-Haas S. Lago-Penas C. Chamari K. (2011). Small-sided games in soccer: amateur vs profesional players’ physiological responses, physical, and technical activities. *J. Strength Cond. Res.* 25 2371–2381. 10.1519/JSC.0b013e3181fb4296 21869625

[B18] Edwards L. C. Bryant A. S. Keegan R. J. Morgan K. Cooper S. M. Jones A. M. (2018). ‘Measuring’ physical literacy and related constructs: a systematic review of empirical findings. *Sports Med.* 48 659–682. 10.1017/s40279-017-0817-9 29143266 PMC5808047

[B19] Edwards S. (1993). *The Heart Rate Monitor Book.* Kempele: Polar CIC

[B20] Elia M. (2013). Body composition by whole-body bioelectrical impedance and prediction of clinically relevant outcomes: overvalued or underused? *Eur. J. Clin. Nutr.* 67 60–70. 10.1038/ejcn.2012.166 23299873

[B21] Faden R. R. Beauchamp T. L. King N. M. P. (1986). *A History and Theory of Informed Consent.* Oxford: Oxford University Press.

[B22] Fitzpatrick J. Hicks K. Hayes P. (2018). Dose-response relationship between training load and changes in aerobic fitness in professional youth soccer players. *Int. J. Sports Physiol. Perform.* 13 1365–1370. 10.1123/ijspp.2017-0843 29745785

[B23] Fredriksen P. M. Skär A. Mamen A. (2018). Waist circumference in 6-12-year-old children: the health oriented pedagogical project (HOPP). *Scand. J. Public Health* 46 12–20. 10.1177/1403494818767790 29754573

[B24] Gäbler M. Prieske O. Hortobágyi T. Granacher U. (2018). The effects of concurrent strength and endurance training on physical fitness and athletic performance in youth: a systematic review and meta-analysis. *Front. Physiol.* 9:1057. 10.3389/fphys.2018.01057 30131714 PMC6090054

[B25] García-Angulo A. García-Angulo F. J. Torres-Luque G. Ortega-Toro E. (2019). Applying the new teaching methodologies in youth football players: toward a healthier sport. *Front. Physiol.* 10:121. 10.3389/fphys.2019.00121 30814956 PMC6381055

[B26] González-Víllora S. Clemente F. M. Lourenço-Martins F. M. Pastor-Vicedo J. C. (2017). Effects of regular and conditioned small-sided games on young football players’ heart rate responses, technical performance and network structure. *Hum. Mov.* 18 135–145. 10.5114/hm.2017.73618

[B27] González-Víllora S. Evangelio C. Sierra-Díaz J. Fernández-Río J. (2018). Hybridizing pedagogical models: a systematic review. *Eur. Phys. Educ. Rev.* 1 1–19. 10.1177/1356336X18797363

[B28] Goodie J. L. Larkin K. T. Schauss S. (2000). Validation of the polar heart rate monitor for assessing heart rate during physical and mental stress. *J. Psychophysiol.* 14 159–164. 10.1027/0269-8803.14.3.159

[B29] Goodyear V. A. Casey A. Kirk D. (2016). Practice architectures and sustainable curriculum renewal. *J. Curric. Stud.* 49 235–254. 10.1080/00220272.2016.1149223

[B30] Gray S. Sproule J. (2011). Developing pupils’ performance in team invasion games. *Phys. Educ. Sport Pedag.* 16 15–32. 10.1080/17408980903535792

[B31] Haddad M. Stylianides G. Djaoui L. Dellal A. Chamari K. (2017). Session-RPE method for training load monitoring: validity, ecological usefulness, and influencing factors. *Front. Neurosci.* 11:612. 10.3389/fnins.2017.00612 29163016 PMC5673663

[B32] Haerens L. Kirk D. Cardon G. De-Bourdeaudhuij I. (2011). Toward the development of a pedagogical model for health-based physical education. *Quest* 63 321–338. 10.1080/00336297.2011.10483684

[B33] Halouani J. Chtourou H. Dellal A. Chaouachi A. Chamari K. (2014). Physiological responses according to rules changes during 3 vs. 3 Small-Sided Games in youth soccer players: stop-ball vs. Small-goals rules. *J. Sports Sci.* 32 1485–1490. 10.1080/02640414.2014.899707 24716549

[B34] Harvey S. Jarrett K. (2014). A review of the game-centred approaches to teaching and coaching literature since 2006. *Phys. Educ. Sport Pedag.* 19 278–300. 10.1080/17408989.2012.754005

[B35] Harvey S. Pill S. Almond L. (2018). Old wine in new bottles: a response to claims that teaching games for understanding was not developed as a theoretically based pedagogy framework. *Phys. Educ. Sport Pedag.* 23 166–180. 10.1080/17408989.2017.1359526

[B36] Heishman A. Curts M. A. Salba E. Hornett R. J. Malin S. K. Weltman A. L. (2018). Noninvasive assessment of internal and external player load: implications for optimizing athletic performance. *J. Strength Cond. Res.* 32 1280–1287. 10.1519/JSC.0000000000002413 29373427

[B37] Hernández-Sampieri R. (2016). *Fundamentos de Investigación.* Spain: McGraw-Hill.

[B38] Hill-Haas S. Dawson B. Impellizzeri F. M. Coutts A. J. (2011). Physiology of small-sided games training in football. A systematic review. *Sports Med.* 41 199-220. 10.2165/11539740-000000000-00000 21395363

[B39] Hoff J. Wisløff W. Engen L. C. Kemi O. J. Helgerud J. (2002). Soccer specific aerobic endurance training. *Br. J. Sports Med.* 36 218–221. 10.1136/bjsm.36.3.218 12055120 PMC1724499

[B40] Holfelder B. Schott N. (2014). Relationship of fundamental movement skills and physical activity in children and adolescents: a systematic review. *Psychol. Sport Exerc.* 15 382–391. 10.1016/j.psychsport.2014.03.0051469-0292

[B41] Hopkins W. G. Marshall S. W. Batterham A. M. Hanin J. (2009). Progressive statistics for studies in sports medicine and exercise science. *Med. Sci. Sports Exerc.* 41 3–13. 10.1249/MSS.0b013e31818cb278 19092709

[B42] Impellizzeri F. M. Rampinini E. Marcora S. M. (2005). Physiological assessment of aerobic training in soccer. *J. Sports Sci.* 23 583–592. 10.1080/02640410400021278 16195007

[B43] Jaspers A. Beéck T. Brink M. S. Frencken W. G. Staes F. Davis J. J. (2018). Relationships between the external and internal training load in professional soccer: what can we learn from machine learning? *Int. J. Sports Physiol. Perform.* 13 625–630. 10.1123/ijspp.2017-0299 29283691

[B44] Khambalia A. Z. Dickinson S. Hardy L. L. Gill T. Baur L. A. (2012). A synthesis of existing systematic reviews and meta-analyses of school-based behavioural interventions for controlling and preventing obesity. *Obes. Rev.* 13 214–233. 10.1111/j.1467-789X.2011.00947.x 22070186

[B45] Kirk D. (2017). Teaching games in physical education: towards a pedagogical model. *RPCD* 17 17–26. 10.5628/rpcd.17.S1A.17

[B46] Kirk D. Macdonald D. O’Sullivan M. (2006). *The handbook of Physical Education.* London: SAGE Publications.

[B47] Kolovelonis A. Goudas M. (2018). The relation of physical self-perceptions of competence, goal orientation, and optimism with students’ performance calibration in Physical Education. *Learn. Individ. Differ.* 61 77–86. 10.1016/j.lindif.2017.11.013

[B48] Landis J. R. Kock G. C. (1977). The measurement of observer agreement for categorical data. *Biometrics* 33 159–174. 10.2307/2529310 843571

[B49] Lang J. J. Tomkinson G. R. Janssen I. Ruiz J. R. Ortega F. B. Léger L. (2018). Making a case for cardiorespiratory fitness surveillance among children and youth. *Exerc. Sport Sci. Rev.* 46 66–75. 10.1249/JES.0000000000000138 29346159

[B50] Léger L. A. Mercier D. Gadoury C. Lambert J. (1988). The multistage 20 metre shuttle run test for aerobic fitness. *J. Sports Sci.* 6 93–101. 10.1080/02640418808729800 3184250

[B51] Li W. Xie X. Li H. (2018). Situated game teaching through set plays: a curricular model to teaching sports in Physical Education. *J. Teach. Phys. Educ.* 37 352–362. 10.1123/jtpe.2018-0001

[B52] Malone J. J. Lovell R. Varley M. C. Coutts A. J. (2017). Unpacking the black box: applications and considerations for using GPS devices in sport. *Int. J. Sports Physiol. Perform.* 12 218–226. 10.1123/ijspp.2016-0236 27736244

[B53] Martín-Martínez I. Reigal-Garrido R. Chirosa-Ríos L. J. Hernández-Mendo A. Chirosa-Ríos I. Martín-Tamayo I. (2015). Effects of a small-sided games program on rate of perceived exertion in a simple of teenage girls. *Cuad. Psico. Deporte* 15 89–98.

[B54] McLaren S. J. Macpherson T. M. Coutts A. J. Hurst C. Spears I. R. Weston M. (2017). The relationships between internal and external measures of training load and intensity in team sports: a meta-analysis. *Sports Med.* 48 641–658. 10.1007/s40279-017-0830-z 29288436

[B55] Metzler M. W. (2017). *Instructional Models in Physical Education.* Routledge: Taylor & Francis.

[B56] Molina-Carmona I. Gómez-Carmona C. D. Bastida-Castillo A. Pino-Ortega J. (2018). Validity of WIMU PRO^TM^ inertial device to register heart rate variable in a field test. *SporTK* 7 81–86. 10.6018/321921

[B57] Mooi E. Sarstedt M. (2011). *A Concise Guide to Market Research: The Process, Data, and Methods Using IBM SPSS Statistics.* Berlin: Springer.

[B58] Morales-Belando M. T. Calderón A. Arias-Estero J. L. (2018). Improvement in game performance and adherence after an aligned TGfU floorball unit in physical education. *Phys. Educ. Sport Pedag.* 23 657–671. 10.1080/17408989.2018.1530747

[B59] Nathan S. (2017). The effect of Teaching Games of Understanding as a coaching instruction had on adjust, cover and heart rate among Malaysian and Indian junior hockey players. *Sports* 44 2–14. 10.3390/sports5020044 29910404 PMC5968981

[B60] Nuttall F. Q. (2015). Obesity, BMI, and health: a critical review. *Nutr. Today* 3 117–128. 10.1097/NT.0000000000000092 27340299 PMC4890841

[B61] Olthof S. Frencken W. Lemmink K. (2018). Match-derived relative pitch area changes the physical and team tactical performance of elite soccer players in Small-Sided soccer games. *J. Sports Sci.* 36 1557–1563. 10.1080/02640414.2017.1403412 29125029

[B62] Patterson J. Hanzel O. Shryack G. Willoughby C. Smith B. (2018). Comparison of two-heart rate technologies to predict VO2max. *Med. Sci. Sports Exerc.* 49:674 10.1249/01.mss.0000538223.86529.62

[B63] Paulson T. A. Mason B. Rhodes J. Goosey-Tolfrey V. L. (2015). Individualized internal and external training load relationships in elite wheelchair rugby players. *Front. Physiol.* 6:388. 10.3389/fphys.2015.00388 26733881 PMC4685065

[B64] Pill S. (2016). An appreciative inquiry exploring game sense teaching in physical education. *Sport Educ. Soc.* 21 279–297. 10.1080/13573322.2014.912624

[B65] Pind R. Mäestu J. (2017). Monitoring training load: necessity, methods and applications. *Acta Kinesiol. Univers. Tartuensis* 23 7–18. 10.12697/akut.2017.23.01

[B66] Pinto D. (2012). Clinical procedures used for analysis of the body composition. *Braz. J. Kineanthropometry Hum. Perform.* 15 113–129. 10.5007/1980-0037.2013v15n1p113

[B67] Práxedes A. Moreno A. Sevil J. García-González L. Del-Villar F. (2016). A preliminary study of the effects of a comprehensive teaching program, based on questioning, to improve tactical actions in young footballers. *Percept. Mot. Skills* 122 742–756. 10.1177/0031512516649716 27207601

[B68] Rocamora I. González-Víllora S. Fernández-Río J. Arias-Palencia N. M. (2019). Physical activity levels, game performance and friendship goals using two different pedagogical models: Sport Education and Direct Instruction. *Phys. Educ. Sport Pedag.* 24 87–102. 10.1080/17408989.2018.1561839

[B69] Rojas-Inda S. (2018). Analysis of internal and external load in small games in young football players. *Rev. Int. Med. Cienc. Act. Fis Deporte* 18 463–477. 10.15366/rimcafd2018.71.004 24589469

[B70] Ruiz J. R. España-Romero V. Castro-Piñero J. Artero E. G. Ortega F. B. Cuenca M. (2011). Alpha-fitness test battery: health-related field-based fitness tests assessment in children adn adolescents. *Nutr. Hosp.* 26 1210–1214. 10.3305/nh.2011.26.6.5270 22411362

[B71] Sanders D. Abt G. Hesselink M. K. Myers T. Akubat I. (2017). Methods of monitoring training load and their relationships to changes in fitness and performance in competitive road cyclists. *Int. J. Sports Physiol. Perform.* 12 668–675. 10.1123/ijspp.2016-0454 28095061

[B72] Scanlan A. T. Wen N. Tucker P. S. Dalbo V. J. (2014). The relationships between internal and external training load models during basketball training. *J. Strength Cond. Res.* 28 2397–2405. 10.1519/jsc.0000000000000458 24662233

[B73] Schulz K. F. Altman D. G. Moher D. (2010). CONSORT 2010 statement: updated guidelines for reporting parallel group randomised trials. *Br. Med. J.* 340 698–702. 10.1136/bmj.c332 21350618 PMC3043330

[B74] Scott D. Lovell R. (2017). Individualisation of speed thresholds does not enhance the dose-response determination in football training. *J. Sports Sci.* 36 1523–1532. 10.1080/02640414.2017.1398894 29099673

[B75] Silva-Arantes A. (2018). Education, medicine and racialization in classrooms of Physical Education of Primary schools (Pernambuco, decade of 1930). *Hist. Educ.* 22 246–262. 10.1590/2236-3459/67595

[B76] Stolz S. Pill S. (2014). Teaching games and sport for understanding: exploring and reconsidering its relevance in Physical Education. *Eur. Phys. Educ. Rev.* 20 36–71. 10.1177/1356336X13496001

[B77] Sullivan G. M. Feinn R. (2012). Using effect size- or why the *p* value is not enough. *J. Graduate Educ.* 4 279–282. 10.4399/JGME-D-12-00156.1 PMC3444174 23997866

[B78] Tavares F. (2015). *Jogos Desportivos Colectivos: Ensinar A Jogar.* Portugal: Editora.

[B79] Thompson B. (2007). Effect sizes, confidence intervals, and confidence intervals for effect size. *Psychol. Schools* 44 423–432. 10.1002/pits.20234

[B80] Utesch T. Dreiskämper D. Naul R. Geukes K. (2018). Understanding physical (in-) activity, overweight, and obesity in childhood: effects of congruence between physical self-concept and motor competence. *Sci. Rep.* 8 1–10. 10.1038/s41598-018-24139-y 29651046 PMC5897370

[B81] Vanrenterghem J. Nedergaard N. J. Robinson M. A. Drust B. (2017). Training load monitoring in team sports: a novel framework separating physiological and biomechanical load-adaptation pathways. *Sports Med.* 47 2135–2142. 10.1007/s40279-017-0714-2 28283992

[B82] World Medical Association (2013). *WMA Declaration of Helsinki – Ethical Principles for Medical Research Involving Human Subjects.* Available at: https://www.wma.net/ (accessed April 13, 2018).

