# Supplementary material for: The Way to Increase the Motor and Sport Competence Among Children: The Contextualized Sport Alphabetization Model
Source: Front Physiol. 2019 May 16;10:569. doi: 10.3389/fphys.2019.00569 (PMC6532438; doi:10.3389/fphys.2019.00569)
Supplement: Supplementary file 1 [file Data_Sheet_1.PDF]

Table 1. Analysis of external and internal TLs among male participants.

Male participants						
	**TGfU group**		**CSAM group**		***p***	***d***
	**Means**	**SD**	**Means**	**SD**		
**Distance covered**	833.92	159.99	1483.84	188.67	< 0.001	3.69
**m·m^{-1}**	36.43	14.36	53.98	18.31	< 0.001	1.05
**Maximum speed**	20.19	2.76	23.97	2.38	< 0.001	1.47
**Average speed**	2.56	0.74	3.55	1.08	< 0.001	1.05
**Number of sprints**	6.01	1.09	14.30	2.96	< 0.001	3.61
**Maximum HR**	189.34	7.52	206.19	5.63	< 0.001	2.56
**Minimum HR**	98.26	11.75	91.84	7.93	< 0.001	0.64
**Average HR**	149.30	8.04	180.11	8.77	< 0.001	3.65
**Edwards' TRIMP**	10.58	2.64	22.00	1.53	< 0.001	5.39
**Calories Burned**	90.58	26.07	143.37	44.33	< 0.001	1.42

Table 2. Pre-test analysis of external and internal TLs among male participants.

Male participants							
**ANOVA 4 vs 4 SSCGs Pre-test**							
	**TGfU group**		**CSAM group**		***F***	***p***	***d***
	**Means**	**SD**	**Means**	**SD**			
**Distance covered**	187.94	47.96	185.44	48.48	0.04	0.834	-
**m·m^{-1}**	17.77	3.17	17.11	3.36	0.68	0.412	-
**Maximum speed**	16.23	1.15	16.13	0.94	0.14	0.707	-
**Average speed**	2.17	4.08	1.50	0.50	0.97	0.327	-
**Number of sprints**	0.90	0.79	0.69	0.78	1.17	0.283	-
**Maximum HR**	98.32	9.83	98.58	8.05	0.01	0.905	-
**Minimum HR**	68.58	10.81	68.67	11.73	0.01	0.975	-
**Average HR**	82.74	9.07	83.06	9.55	0.01	0.891	-
**Edwards' TRIMP**	11.00	3.51	11.47	3.41	0.31	0.579	-
**Calories Burned**	31.74	10.63	31.25	9.97	0.03	0.846	-
**VO2 max**	48.19	3.69	48.36	2.99	0.04	0.841	-

Table 3. Post-test analysis of external and internal TLs among male participants.

Male participants							
**ANCOVA 4 vs 4 SSCGs Post-test**							
	**TGfU group**		**CSAM group**		***F***	***p***	***d***
	**Means**	**SD**	**Means**	**SD**			
**Distance covered**	260.61	50.97	317.75	50.66	58.09	< 0.001	1.12
**m·m^{-1}**	19.58	3.09	24.69	2.95	133.18	< 0.001	1.69
**Maximum speed**	17.81	1.84	24.13	2.10	247.66	< 0.001	3.18
**Average speed**	2.41	4.01	2.55	0.32	109.97	< 0.001	0.05
**Number of sprints**	4.10	2.97	9.25	3.85	68.50	< 0.001	1.48
**Maximum HR**	122.26	15.88	151.78	11.93	137.64	< 0.001	2.12
**Minimum HR**	86.48	10.25	101.25	10.40	196.29	< 0.001	1.42
**Average HR**	105.52	15.24	121.50	14.03	57.46	< 0.001	1.09
**Edwards' TRIMP**	10.58	2.64	22.00	1.53	467.21	< 0.001	5.39
**Calories Burned**	41.94	13.86	54.94	13.68	108.19	< 0.001	0.94
**VO2 max**	55.42	6.16	68.29	3.51	174.27	< 0.001	2.61

Table 4. Analysis of external and internal TLs among female participants.

Female participants						
	**TGfU group**		**CSAM group**		***p***	***d***
	**Means**	**SD**	**Means**	**SD**		
**Distance covered**	717.73	121.51	1316.88	212.72	< 0.001	3.39
**m·m^{-1}**	26.78	9.23	49.23	12.77	< 0.001	1.99
**Maximum speed**	18.22	2.60	22.67	5.05	0.001	1.08
**Average speed**	2.01	0.65	3.21	0.98	< 0.001	1.42
**Number of sprints**	5.89	1.13	13.16	3.45	< 0.001	2.75
**Maximum HR**	187.19	12.14	201.18	9.12	< 0.001	1.31
**Minimum HR**	95.72	9.20	109.88	6.35	< 0.001	1.81
**Average HR**	145.14	9.98	178.59	9.94	< 0.001	3.35
**Edwards' TRIMP**	12.50	3.93	20.10	2.44	< 0.001	2.36
**Calories Burned**	79.33	16.65	141.53	33.37	< 0.001	2.29

Table 5. Pre-test analysis of external and internal TLs among female participants.

Female participants							
**ANOVA 4 vs 4 SSCGs Pre-test**							
	**TGfU group**		**CSAM group**		***F***	***p***	***d***
	**Means**	**SD**	**Means**	**SD**			
**Distance covered**	210.92	59.93	219.24	69.79	0.18	0.669	-
**m·m^{-1}**	18.21	3.83	17.48	3.43	0.45	0.506	-
**Maximum speed**	16.51	1.25	16.29	1.18	0.35	0.555	-
**Average speed**	1.75	0.66	1.62	0.69	0.38	0.540	-
**Number of sprints**	0.79	0.83	0.86	0.79	0.07	0.789	-
**Maximum HR**	103.33	10.26	103.52	11.81	0.01	0.954	-
**Minimum HR**	74.08	12.93	73.62	12.39	0.01	0.903	-
**Average HR**	86.29	10.28	85.43	9.48	0.08	0.772	-
**Edwards' TRIMP**	12.54	3.90	10.71	3.83	2.49	0.121	-
**Calories Burned**	36.21	10.71	34.71	9.76	0.23	0.629	-
**VO2 max**	47.60	3.66	46.20	8.71	0.51	0.477	-

Table 6. Post-test analysis of external and internal TLs among female participants.

Female participants							
**ANCOVA 4 vs 4 SSCGs Post-test**							
	**TGfU group**		**CSAM group**		***F***	***p***	***d***
	**Means**	**SD**	**Means**	**SD**			
**Distance covered**	287.67	54.59	333.29	57.88	24.91	< 0.001	0.80
**m·m^{-1}**	19.92	3.97	26.10	3.47	92.81	< 0.001	1.66
**Maximum speed**	18.74	2.55	24.20	2.84	94.74	< 0.001	2.01
**Average speed**	2.00	0.46	2.54	0.42	39.93	< 0.001	1.22
**Number of sprints**	5.29	2.78	10.71	3.66	78.91	< 0.001	1.65
**Maximum HR**	130.38	15.71	153.95	13.12	52.78	< 0.001	1.63
**Minimum HR**	92.46	14.98	104.57	12.17	16.21	< 0.001	0.89
**Average HR**	106.29	14.47	120.90	13.43	31.90	< 0.001	1.04
**Edwards' TRIMP**	12.50	3.93	20.10	2.44	65.90	< 0.001	2.36
**Calories Burned**	48.29	14.13	60.76	13.57	39.34	< 0.001	0.90
**VO2 max**	51.82	7.77	65.27	3.99	40.05	< 0.001	2.22

Table 7. Analysis of external and internal TLs during the intervention at First grade (U-7).

	First grade (n = 20)				Under 7 years old	
	TGfU group		CSAM group		*p*	*d*
	Means	SD	Means	SD		
**Distance covered**	791.34	248.37	1368.00	207.05	< 0.001	2.52
**m·m^{-1}**	41.45	18.64	72.22	12.41	< 0.001	1.94
**Maximum speed**	19.22	2.14	22.51	2.26	< 0.001	0.20
**Average speed**	2.44	0.86	4.09	0.73	0.006	2.06
**Number of sprints**	5.38	1.01	10.69	1.52	< 0.001	4.11
**Maximum HR**	190.37	10.13	201.95	5.91	0.006	1.39
**Minimum HR**	106.15	12.20	115.56	9.39	0.069	-
**Average HR**	151.64	7.99	167.38	7.58	< 0.001	2.02
**Edwards' TRIMP**	11.00	2.26	21.00	1.88	< 0.001	4.80
**Calories Burned**	65.26	10.44	99.43	10.20	< 0.001	3.31

Table 8. Pre-test analysis of external and internal TLs at First grade (U-7).

	First grade (n = 20)					Under 7 years old	
	ANOVA 4 vs 4 SSCGs Pre-test						
	TGfU group		CSAM group		*F*	*p*	*d*
	Means	SD	Means	SD			
**Distance covered**	155.70	4.90	155.00	4.19	0.11	0.735	-
**m·m^{-1}**	16.70	1.76	16.60	2.06	0.01	0.909	-
**Maximum speed**	16.50	1.14	16.44	1.07	0.01	0.905	-
**Average speed**	0.86	0.27	0.88	0.26	0.02	0.870	-
**Number of sprints**	0.40	0.51	0.20	0.42	0.90	0.355	-
**Maximum HR**	95.20	1.68	95.40	2.27	0.05	0.826	-
**Minimum HR**	53.20	2.48	51.70	3.30	1.31	0.266	-
**Average HR**	67.80	3.08	67.60	3.43	0.01	0.839	-
**Edwards' TRIMP**	11.30	3.94	9.30	2.31	1.91	0.184	-
**Calories Burned**	15.70	2.40	16.40	2.54	0.39	0.535	-
**VO_2 max**	49.88	3.63	49.20	2.06	0.26	0.613	-

Table 9. Post-test analysis of external and internal TLs at First grade (U-7).

	First grade (n = 20)					Under 7 years old	
	ANCOVA 4 vs 4 SSCGs Post-test						
	TGfU group		CSAM group		*F*	*p*	*d*
	Means	SD	Means	SD			
**Distance covered**	186.80	4.75	232.00	14.29	61.06	< 0.001	4.24
**m·m^{-1}**	18.40	1.17	22.30	2.35	10.45	0.014	2.10
**Maximum speed**	18.30	0.91	22.10	1.17	31.34	0.001	3.62
**Average speed**	1.48	0.11	2.13	0.17	180.33	< 0.001	4.53
**Number of sprints**	2.30	1.33	5.80	1.22	104.30	< 0.001	2.74
**Maximum HR**	103.20	6.54	130.40	7.18	83.93	< 0.001	3.96
**Minimum HR**	72.70	2.05	88.50	4.06	132.09	< 0.001	2.90
**Average HR**	84.10	4.19	97.90	10.79	34.50	0.001	1.68
**Edwards' TRIMP**	11.00	2.26	21.00	1.88	54.10	< 0.001	4.81
**Calories Burned**	65.26	10.44	99.43	10.20	41.57	< 0.001	3.31
**VO2 max**	48.88	2.62	63.28	6.13	57.68	< 0.001	3.05

Table 10. Analysis of external and internal TLs during the intervention at Second grade (U-8).

	Second grade (n = 19)				Under 8 years old	
	TGfU group		CSAM group		*p*	*d*
	Means	SD	Means	SD		
**Distance covered**	830.35	139.51	1440.30	112.40	< 0.001	4.84
**m·m^{-1}**	41.45	9.66	65.55	8.48	< 0.001	2.66
**Maximum speed**	21.70	2.62	24.99	6.67	0.185	-
**Average speed**	3.25	0.71	4.21	0.82	0.015	1.24
**Number of sprints**	6.12	0.64	13.75	1.82	< 0.001	5.46
**Maximum HR**	194.62	8.32	206.70	6.30	0.002	1.65
**Minimum HR**	97.99	11.18	109.17	8.10	0.022	0.19
**Average HR**	152.62	5.04	183.30	8.39	< 0.001	4.37
**Edwards' TRIMP**	12.22	3.66	21.30	1.56	< 0.001	3.29
**Calories Burned**	71.17	17.28	130.66	18.90	< 0.001	3.27

Table 11. Pre-test analysis of external and internal TLs at Second grade (U-8).

	Second grade (n = 19)					Under 8 years old	
	ANOVA 4 vs 4 SSCGs Pre-test						
	TGfU group		CSAM group		*F*	*p*	*d*
	Means	SD	Means	SD			
**Distance covered**	148.89	4.13	149.50	4.95	0.08	0.775	-
**m·m^{-1}**	14.22	1.20	14.20	1.03	0.01	0.966	-
**Maximum speed**	14.56	0.45	14.51	0.44	0.07	0.787	-
**Average speed**	1.23	0.15	1.29	0.11	0.83	0.373	-
**Number of sprints**	0.78	1.30	0.60	1.07	0.10	0.748	-
**Maximum HR**	93.11	1.53	93.60	1.07	0.65	0.429	-
**Minimum HR**	65.55	1.23	65.50	0.97	0.01	0.914	-
**Average HR**	79.56	1.13	79.60	1.07	0.01	0.931	-
**Edwards' TRIMP**	11.00	2.95	10.30	2.66	0.29	0.595	-
**Calories Burned**	30.22	2.10	27.20	2.70	7.27	0.015	-
**VO2 max**	49.47	2.28	18.06	2.00	2.08	0.167	-

Table 12. Post-test analysis of external and internal TLs at Second grade (U-8).

	Second grade (n = 19)					Under 8 years old	
	ANCOVA 4 vs 4 SSCGs Post-test						
	TGfU group		CSAM group		*F*	*p*	*d*
	Means	SD	Means	SD			
**Distance covered**	281.67	4.69	320.20	6.39	34.03	0.001	6.81
**m·m^{-1}**	15.89	1.45	23.50	2.41	25.74	0.002	3.77
**Maximum speed**	16.68	1.12	22.63	0.49	74.31	< 0.001	7.02
**Average speed**	1.58	0.21	2.61	0.19	36.88	0.001	5.18
**Number of sprints**	3.11	1.90	8.10	0.73	5.17	0.063	-
**Maximum HR**	130.22	1.30	147.60	2.63	36.57	0.001	8.23
**Minimum HR**	86.00	19.87	93.20	1.84	0.25	0.632	-
**Average HR**	93.33	5.72	117.00	12.93	3.29	0.120	2.32
**Edwards' TRIMP**	12.22	3.66	21.30	1.56	14.07	0.009	3.29
**Calories Burned**	71.17	17.28	130.66	18.90	13.98	0.010	3.27
**VO2 max**	53.91	5.27	67.78	2.08	4.97	0.067	3.53

Table 13. Analysis of external and internal TLs during the intervention at Third grade (U-9).

	Third grade (n = 19)				Under 9 years old	
	TGfU group		CSAM group		*p*	*d*
	Means	SD	Means	SD		
**Distance covered**	794.32	134.39	1447.02	193.61	0.000	3.87
**m·m^{-1}**	30.40	6.04	40.05	4.00	0.001	1.90
**Maximum speed**	19.89	2.45	23.50	3.25	0.015	1.24
**Average speed**	2.24	0.23	2.72	1.17	0.240	-
**Number of sprints**	5.62	1.11	12.16	2.15	< 0.001	3.74
**Maximum HR**	190.32	9.60	207.60	6.13	< 0.001	2.17
**Minimum HR**	88.80	8.90	107.85	4.10	< 0.001	2.80
**Average HR**	148.71	9.33	181.68	3.21	< 0.001	4.83
**Edwards' TRIMP**	8.67	1.22	21.60	1.57	< 0.001	9.13
**Calories Burned**	87.31	14.15	140.82	8.62	< 0.001	4.63

Table 14. Pre-test analysis of external and internal TLs at Third grade (U-9).

	Third grade (n = 19)					Under 9 years old	
	ANOVA 4 vs 4 SSCGs Pre-test						
	TGfU group		CSAM group		*F*	*p*	*d*
	Means	SD	Means	SD			
**Distance covered**	156.89	5.77	154.20	5.24	1.13	0.302	-
**m·m^{-1}**	16.89	1.45	15.50	1.58	3.94	0.063	-
**Maximum speed**	15.84	0.67	15.97	0.32	0.27	0.607	-
**Average speed**	1.56	0.15	1.57	0.18	0.01	0.966	-
**Number of sprints**	0.78	0.83	0.60	0.69	0.25	0.620	-
**Maximum HR**	94.78	1.71	97.40	2.41	0.15	0.702	-
**Minimum HR**	64.78	1.09	65.30	2.00	0.48	0.497	-
**Average HR**	83.22	1.00	84.10	0.99	0.89	0.357	-
**Edwards' TRIMP**	10.22	2.53	9.80	2.89	0.11	0.741	-
**Calories Burned**	31.00	2.29	31.60	1.95	0.37	0.546	-
**VO2 max**	46.72	3.88	48.14	3.34	0.73	0.404	-

Table 15. Post-test analysis of external and internal TLs at Third grade (U-9).

	Third grade (n = 19)					Under 9 years old	
	ANCOVA 4 vs 4 SSCGs Post-test						
	TGfU group		CSAM group		*F*	*p*	*d*
	Means	SD	Means	SD			
**Distance covered**	294.44	4.77	325.70	4.27	94.55	< 0.001	6.92
**m·m^{-1}**	19.00	0.86	24.00	0.94	64.74	< 0.001	5.53
**Maximum speed**	16.71	0.60	24.06	1.92	162.13	< 0.001	5.04
**Average speed**	1.72	0.10	2.32	0.19	33.70	0.001	3.88
**Number of sprints**	1.67	1.32	6.40	1.83	15.26	0.008	2.93
**Maximum HR**	116.67	13.52	151.70	3.36	31.68	0.001	3.65
**Minimum HR**	85.33	4.77	98.00	1.82	132.00	< 0.001	3.58
**Average HR**	110.56	6.80	127.70	6.84	38.58	0.001	2.51
**Edwards' TRIMP**	8.67	1.22	21.60	1.57	205.46	< 0.001	9.13
**Calories Burned**	87.31	14.15	140.82	8.62	28.00	0.002	4.63
**VO2 max**	58.48	7.22	68.30	2.38	175.48	< 0.001	1.87

Table 16. Analysis of external and internal TLs during the intervention at Fourth grade (U-10).

	**Fourth grade (n = 21)**				**Under 10 years old**	
	**TGfU group**		**CSAM group**		***p***	***d***
	**Means**	**SD**	**Means**	**SD**		
**Distance covered**	741.29	66.70	1290.12	271.65	< 0.001	2.71
**m·m^{-1}**	23.06	4.66	34.12	7.36	0.001	1.77
**Maximum speed**	17.65	1.83	23.35	2.70	< 0.001	2.44
**Average speed**	1.92	0.27	3.62	1.04	< 0.001	2.18
**Number of sprints**	5.84	1.54	13.87	3.45	< 0.001	2.95
**Maximum HR**	180.70	10.96	201.68	11.09	< 0.001	1.90
**Minimum HR**	94.46	6.21	113.76	7.80	< 0.001	2.72
**Average HR**	178.96	10.84	201.68	10.84	< 0.001	2.09
**Edwards' TRIMP**	11.30	2.66	20.73	3.13	< 0.001	3.23
**Calories Burned**	88.18	7.87	128.30	18.74	< 0.001	2.74

Table 17. Pre-test analysis of external and internal TLs at Fourth grade (U-10).

	**Fourth grade (n = 21)**				**Under 10 years old**		
	**ANOVA 4 vs 4 SSCGs Pre-test**						
	**TGfU group**		**CSAM group**		***F***	***p***	***d***
	**Means**	**SD**	**Means**	**SD**			
**Distance covered**	195.40	7.24	208.64	58.05	0.51	0.484	-
**m·m^{-1}**	17.00	1.63	16.64	1.74	0.24	0.629	-
**Maximum speed**	16.55	0.19	16.51	0.16	0.16	0.687	-
**Average speed**	1.39	0.26	1.37	0.20	0.02	0.870	-
**Number of sprints**	1.00	0.66	1.09	0.70	0.09	0.765	-
**Maximum HR**	94.50	5.38	94.64	2.11	0.00	0.939	-
**Minimum HR**	74.80	2.11	74.82	3.31	0.17	0.677	-
**Average HR**	89.00	3.01	88.09	3.72	0.37	0.549	-
**Edwards' TRIMP**	11.40	40.83	12.09	3.61	0.13	0.713	-
**Calories Burned**	36.60	3.59	35.73	3.03	0.36	0.554	-
**VO2 max**	48.36	3.85	46.61	4.00	1.02	0.323	-

Table 18. Post-test analysis of external and internal TLs at Fourth grade (U-10).

	**Fourth grade (n = 21)**				**Under 10 years old**		
	**ANCOVA 4 vs 4 SSCGs Post-test**						
	**TGfU group**		**CSAM group**		***F***	***p***	***d***
	**Means**	**SD**	**Means**	**SD**			
**Distance covered**	246.90	28.34	317.64	30.00	10.76	0.011	2.42
**m·m^{-1}**	18.00	1.63	25.18	2.85	68.71	< 0.001	3.05
**Maximum speed**	17.51	0.53	23.63	2.40	31.38	0.001	3.44
**Average speed**	1.60	0.17	2.68	0.48	50.61	< 0.001	2.93
**Number of sprints**	7.70	0.94	12.09	2.80	15.40	0.004	2.05
**Maximum HR**	130.70	13.54	153.91	6.94	21.90	0.002	2.19
**Minimum HR**	93.30	2.31	109.55	7.25	3.78	< 0.001	2.95
**Average HR**	117.20	8.36	127.27	4.65	15.16	0.005	1.50
**Edwards' TRIMP**	11.30	2.66	20.73	3.13	48.27	< 0.001	3.23
**Calories Burned**	88.18	7.87	128.30	18.74	20.47	0.002	2.74
**VO2 max**	55.56	5.44	68.46	5.86	88.22	< 0.001	0.30

Table 19. Analysis of external and internal TLs during the intervention at Fifth grade (U-11).

	**Fifth grade (n = 15)**				**Under 11 years old**	
	**TGfU group**		**CSAM group**		***p***	***d***
	**Means**	**SD**	**Means**	**SD**		
**Distance covered**	828.95	102.47	1477.71	186.74	< 0.001	4.22
**m·m^{-1}**	30.60	5.42	47.44	9.10	0.001	2.20
**Maximum speed**	18.93	2.20	21.96	2.59	0.031	1.25
**Average speed**	2.26	0.38	2.63	0.48	0.128	-
**Number of sprints**	6.65	0.29	16.31	1.28	< 0.001	10.06
**Maximum HR**	192.45	4.69	206.82	6.34	< 0.001	2.54
**Minimum HR**	99.30	7.00	108.52	5.61	0.014	1.46
**Average HR**	147.39	9.17	186.33	3.71	< 0.001	5.72
**Edwards' TRIMP**	12.00	5.32	21.13	2.35	0.001	2.27
**Calories Burned**	104.96	24.09	177.66	31.36	< 0.001	2.57

Table 20. Pre-test analysis of external and internal TLs at Fifth grade (U-11).

	**Fifth grade (n = 15)**					**Under 11 years old**	
	**ANOVA 4 vs 4 SSCGs Pre-test**						
	**TGfU group**		**CSAM group**		***F***	***p***	***d***
	**Means**	**SD**	**Means**	**SD**			
**Distance covered**	263.86	5.11	260.88	6.28	0.99	0.337	-
**m·m^{-1}**	19.29	2.56	18.88	2.69	0.91	0.768	-
**Maximum speed**	16.82	0.60	16.78	0.68	0.01	0.905	-
**Average speed**	5.31	8.20	2.21	0.16	1.14	0.304	-
**Number of sprints**	1.00	0.57	1.13	0.64	0.15	0.700	-
**Maximum HR**	117.29	4.92	115.00	4.27	0.92	0.353	-
**Minimum HR**	86.71	2.81	86.25	1.83	0.14	0.707	-
**Average HR**	95.71	1.49	95.75	1.75	0.01	0.967	-
**Edwards' TRIMP**	12.00	3.78	11.38	3.42	0.11	0.742	-
**Calories Burned**	46.86	1.95	44.75	2.31	3.57	0.081	-
**VO2 max**	48.94	4.33	49.38	2.60	3.59	0.080	-

Table 21. Post-test analysis of external and internal TLs at Fifth grade (U-11).

	**Fifth grade (n = 15)**					**Under 11 years old**	
	**ANCOVA 4 vs 4 SSCGs Post-test**						
	**TGfU group**		**CSAM group**		***F***	***p***	***d***
	**Means**	**SD**	**Means**	**SD**			
**Distance covered**	289.86	43.00	377.75	17.51	4.48	0.169	-
**m·m^{-1}**	22.86	2.96	27.88	1.55	0.01	0.952	-
**Maximum speed**	17.84	2.98	26.70	1.28	24.25	0.039	3.96
**Average speed**	5.37	8.21	2.81	0.14	13.99	0.065	-
**Number of sprints**	8.43	2.50	15.00	0.75	4.62	0.164	-
**Maximum HR**	130.29	9.77	151.87	4.82	0.79	0.467	-
**Minimum HR**	99.00	0.29	112.75	2.05	24.44	0.039	9.06
**Average HR**	120.29	9.23	135.38	2.87	0.09	0.790	-
**Edwards' TRIMP**	12.00	5.32	21.13	2.35	0.32	0.626	-
**Calories Burned**	104.96	24.09	177.66	31.36	376.94	0.003	2.46
**VO2 max**	56.90	10.90	67.66	4.00	0.050	0.835	-

Table 22. Analysis of external and internal TLs during the intervention at Sixth grade (U-12).

Sixth grade (n = 18)						
	TGfU group		CSAM group		*p*	*d*
	Means	SD	Means	SD		
**Distance covered**	732.62	167.38	1563.30	202.54	< 0.001	4.52
**m·m^{-1}**	26.55	15.08	53.61	12.82	0.001	1.91
**Maximum speed**	18.84	4.02	24.36	1.33	0.002	1.75
**Average speed**	1.87	0.83	3.07	0.75	0.006	1.50
**Number of sprints**	6.40	1.16	17.77	2.42	< 0.001	6.24
**Maximum HR**	184.13	6.91	201.51	4.96	< 0.001	2.83
**Minimum HR**	96.12	10.53	115.55	4.13	< 0.001	2.32
**Average HR**	138.52	6.50	181.42	4.13	< 0.001	7.67
**Edwards' TRIMP**	13.30	3.36	22.25	1.83	< 0.001	3.20
**Calories Burned**	101.65	30.13	198.94	51.14	< 0.001	2.39

Table 23. Pre-test analysis of external and internal TLs at Sixth grade (U-12).

	Sixth grade (n = 18)				Under 12 years old		
	ANOVA 4 vs 4 SSCGs Pre-test						
	TGfU group		CSAM group		*F*	*p*	*d*
	Means	SD	Means	SD			
**Distance covered**	277.80	30.92	288.88	4.15	1.00	0.332	-
**m·m^{-1}**	23.60	1.83	23.25	2.37	0.12	0.729	-
**Maximum speed**	17.76	0.71	17.25	0.20	3.74	0.071	-
**Average speed**	2.47	0.14	2.25	0.79	0.74	0.401	-
**Number of sprints**	1.20	0.63	1.00	0.75	0.37	0.549	-
**Maximum HR**	11.90	7.38	116.00	1.51	2.35	0.144	-
**Minimum HR**	84.90	2.33	85.00	1.60	0.01	0.919	-
**Average HR**	92.90	2.84	92.00	3.70	0.34	0.567	-
**Edwards' TRIMP**	14.00	3.55	15.00	4.14	0.30	0.589	-
**Calories Burned**	45.10	1.72	43.88	1.88	2.06	0.170	-
**VO2 max**	46.67	2.96	43.66	13.74	0.45	0.508	-

Table 24. Post-test analysis of external and internal TLs at Sixth grade (U-12).

	Sixth grade (n = 18)				Under 12 years old		
	ANCOVA 4 vs 4 SSCGs Post-test						
	TGfU group		CSAM group		*F*	*p*	*d*
	Means	SD	Means	SD			
**Distance covered**	343.2	6.12	392.87	5.46	459.81	< 0.001	8.50
**m·m^{-1}**	24.70	2.11	29.88	0.83	12.33	0.017	3.09
**Maximum speed**	21.83	1.02	26.92	1.59	49.91	0.001	3.91
**Average speed**	2.48	0.12	2.85	0.14	34.10	0.002	2.86
**Number of sprints**	5.20	0.78	12.75	2.25	24.89	0.004	4.72
**Maximum HR**	144.60	3.50	176.50	7.07	26.24	0.004	5.94
**Minimum HR**	100.50	2.79	117.13	1.24	88.74	< 0.001	7.39
**Average HR**	113.20	5.18	125.50	3.46	20.08	0.007	2.72
**Edwards' TRIMP**	13.30	3.36	22.25	1.83	12.11	0.018	3.16
**Calories Burned**	101.65	30.13	198.94	51.14	5.08	0.074	2.39
**VO2 max**	50.77	6.59	67.67	2.88	46.14	0.001	3.19
